# Simple Rules for Climate Policy and Integrated Assessment

**DOI:** 10.1007/s10640-018-0280-6

**Published:** 2018-08-18

**Authors:** Frederick van der Ploeg, Armon Rezai

**Affiliations:** 1 Department of Economics, OxCarre, University of Oxford, Manor Road Building, Oxford, OX1 3UQ UK; 2 Vrije Universiteit Amsterdam, Amsterdam, The Netherlands; 3 Department of Socioeconomics, Vienna University of Economics and Business, Welthandelsplatz 1, 1020 Vienna, Austria; 4 IIASA, Schlossplatz 1, 2361 Laxenburg, Austria; 5 The Vienna Institute for International Economic Studies, Rahlgasse 3, 1060 Vienna, Austria

**Keywords:** Simple rules, Climate policy, Ethics, Economics, Geophysics, Politics, Discounting with declining discount rates, Positive feedback, Free riding, D81, H20, Q31, Q38

## Abstract

A simple integrated assessment framework that gives rules for the optimal carbon price, transition to the carbon-free era and stranded carbon assets is presented, which highlights the ethical, economic, geophysical and political drivers of optimal climate policy. For the ethics we discuss the role of intergenerational inequality aversion and the discount rate, where we show the importance of lower discount rates for appraisal of longer run benefit and of policy makers using lower discount rates than private agents. The economics depends on the costs and rates of technical progress in production of fossil fuel, its substitute renewable energies and sequestration. The geophysics depends on the permanent and transient components of atmospheric carbon and the relatively fast temperature response, and we allow for positive feedbacks. The politics stems from international free-rider problems in absence of a global climate deal. We show how results change if different assumptions are made about each of the drivers of climate policy. Our main objective is to offer an easy back-on-the-envelope analysis, which can be used for teaching and communication with policy makers.

## Introduction

Our aim is to present a back-on-the-envelope integrated assessment framework that can be used to derive optimal climate policies in a transparent and intuitive way. Climate policy has to deal with several intertemporal, geophysical, and interregional aspects.

To discuss these issues, we use a framework consisting of an economic part (to describe the use of fossil fuel use and its substitute renewable energy, carbon sequestration with trend growth and sector-specific rates of technical progress, global damages to economic production) and a climate part (to describe the dynamics of atmospheric carbon and global mean temperature). This framework allows us to derive welfare-maximising climate policies as simples rules for the optimal carbon price (equal to the social cost of carbon), the rate at which renewable energies are substituted for fossil fuel, the fraction of fossil that is abated by carbon capture and sequestration (CCS), the optimal timing of the transition to the carbon-free era, the maximum cumulative emissions (or the carbon budget for short) and the maximum warming level, and the amount of fossil fuel locked up forever in the crust of the earth. The geophysical, ethical and economic drivers of climate policy can thus clearly be identified.

We highlight various features. Regarding the ethics of climate policy, we allow discount rates to decline with the horizon at which costs and benefits are evaluated. Since the costs of global warming occur many decades or even centuries into the future, this has important implications for policy. This feature is known as hyperbolic discounting and has been put forward by Laibson (1997). Following von Below (2012), Schmitt (2014), Belfiori (2017), and Barrage (2018) we also allow policy makers to have a lower ethical discount rate than the market. Both these features allow us to take a stance between the low discount rate used by Stern (2007) and the high discount rate used by Nordhaus (2008): policy makers use lower discount rates for long-run than for short-run appraisal of costs and benefits *and* may be more farsighted than the market. Both features generally lead to time inconsistency. Given simplifying assumptions, problems of commitment do not arise in our model.^1^ Regarding the geophysical drivers of climate policy, apart from our benchmark of simple linear carbon and temperature dynamics used by atmospheric physicists (e.g., Joos et al. 2013; Allen 2016; Aengenheyster et al. 2018) and economists (e.g., Hassler and Krusell 2012; Golosov et al. 2014; van den Bijgaart et al. 2016; Rezai and van der Ploeg 2016; Gerlagh and Liski 2018), we also allow for a model of carbon dynamics with the positive feedback loop that get unleashed as the capacity of the oceans to absorb carbon diminishes (Millar et al. 2017). Finally, regarding the political drivers of climate policy, we extend our simple rules to allow for non-cooperative decision making to illustrate the point of international free riding and the less ambitious climate policies that result from this (Barrett 2003). This addresses the problem of free riding and is relevant as long as there are no international climate deals with appropriate international transfers to ensure that the global carbon price indeed gets implemented throughout the world economy.

Our objective is not to present any novel theoretical results, but to present a simple framework that is consistent with a large and sometimes hard to comprehend integrated assessment literature. We have used our framework for undergraduate and graduate teaching and in discussions with policy makers and interested lay persons. We have found it useful to highlight the drivers on climate policy and to illustrate various assumptions regarding the ethics, economics, geophysics and politics underlying climate policy.^2^

Our contribution ties in with the emerging literature on simple and robust rules for the optimal carbon price (e.g., Nordhaus 1991; Golosov et al. 2014; Rezai and van der Ploeg 2016; van den Bijgaart et al. 2016; Allen 2016; Dietz and Venmans 2018; van der Ploeg 2018; van den Bremer and van der Ploeg 2018). We also offer simple rules for the optimal transition time to the carbon-free era and the amount of locked up fossil fuel. These simple rules take advantage of the much faster convergence of Ramsey economic growth dynamics than that of the carbon cycle, thus greatly simplifying the complexity of the underlying system. The resulting rules are easy to understand, calculate, explain, and communicate. Furthermore, being simple feedback rules, they appear robust to different model specifications as they perform well in a wide variety of integrated assessment models (Rezai and van der Ploeg 2016; van den Bijgaart et al. 2016; Barrage 2014).

A multitude of very large and detailed Integrated Assessment Models (IAMs) of the economy and the climate are able to generate numerical simulations of the optimal global price of carbon, the implied optimal substitution rates of renewable energies for fossil fuel, and the optimal sequestration rates. Although such IAMs give careful suggestions for climate policies, the key determinants of these are difficult to understand. Furthermore, it has been argued that in providing exact numbers they appear more precise than the underlying science would permit and misrepresent the deep uncertainties surrounding global warming damages and the social cost of carbon (e.g., Pindyck 2013; Wagner and Weitzman 2015; Stern 2016). We therefore prefer a clear and transparent approach in which all the drivers of climate policy are immediately apparent. To our benefit, recent insights in atmospheric science suggest that global warming is well explained by cumulative carbon emissions rather than the stock of carbon in the atmosphere (e.g., Allen 2016), even though large-scale IAMs have sophisticated and high-dimensional models to describe the carbon cycle and temperature responses to emission impulses.

Our back-on-the-envelope IAM is adapted from the most widely-used IAM, i.e., DICE (Dynamic integrated model of climate and the economy; Nordhaus 2008, 2014). In this IAM economic activity requires energy in production which in turn is generated using a continuum of technologies and energy sources. The energy mix with the lowest unit costs use fossil energy use only and have the largest amount of carbon emission per unit of energy. As more renewable energies are substituted for fossil fuel, the cost per unit of energy becomes more expensive whilst the carbon emissions per unit of energy fall. This substitution is driven by a spectrum of carbon-free technologies, ranging from energy-saving to renewable energy generation in combination with gas-fired power plants. The most expensive fuel mix is fully carbon-free and is referred to as the “backstop” technology. Given our current technological knowledge, one can think of this backstop as CCS which takes carbon directly out of the atmosphere when using fossil fuel and then stores it underground as the most carbon-persistent production processes (such as metallurgical ones or air travel) cannot be decarbonised at current capabilities. We thus make explicit the difference between *substitution of less carbon*-*intensive fuel in the energy mix*^3^ and *carbon capture and sequestration*. This distinction is important as both instruments differ in their long-term effects: renewable energy create a legacy of unused fossil fuel deposits which can become economically lucrative if future policy becomes less ambitious while CCS bear the risk of leakage (Belfiori and Iverson 2018).

Like DICE, our benchmark IAM computes cumulative carbon use and does not speak to the issue of stranded carbon assets directly. However, we include an extension where the cost of extracting fossil fuel rises as less reserves are left in situ, which allows the economic analysis of stranded carbon assets too. We also give extensions to allow for research and development in renewable energy production and for CCS becoming more expensive as available CO_2_ reservoirs are being used up.^4^

We thus present a back-of-the-envelope IAM and derive simple rules for the optimal carbon price and climate policies. Section 2 sets up the model. Section 3 derives our simple rule for the optimal price of carbon and the optimal rates of substituting renewable energies for fossil fuel and of CCS. Section 4 discusses the timing of energy transitions, carbon budgets, and peak warming for different policy regimes arising under the optimal climate policy. Section 5 presents the optimal climate policies for our benchmark calibration. Sections 6, 7, 8 and 9 discuss the sensitivity of optimal climate policies to different assumptions regarding the ethical, economic, geophysical and political drivers of climate policies, respectively. In particular, we allow for hyperbolic discounting and positive feedbacks resulting from capacity for absorbing CO_2_ diminishing as the oceans heat up. Section 10 concludes.

## A Back-of-the-Envelope Integrated Assessment Model

Most IAMs simultaneously model the economic dynamics of the productive capabilities and the evolution of the climate. Following earlier work on simple rules (e.g., Nordhaus 1991; Golosov et al. 2014; Rezai and van der Ploeg 2016; van den Bijgaart et al. 2016; Allen 2016; Dietz and Venmans 2018; van der Ploeg 2018; van den Bremer and van der Ploeg 2018), we suppose that the dynamics of economic growth converge much faster than that of the carbon cycle and temperature dynamics. Given this and the long horizons involved in assessing optimal climate policy, we abstract from capital formation and assume for purposes of calculating the social cost of carbon that the economy has converged to its balanced growth path where aggregate global output of goods and services before climate damages, denoted by *Y*, and aggregate global consumption, denoted by *C*, are both growing at the exogenous rate of economic growth, *g*.

Following DICE, we suppose that production of $Y_{t}$ at time *t* requires energy in a fixed and declining proportion, so that global aggregate energy use is $\gamma_{0} e^{{ - r_{\gamma } t}} Y_{t} ,$ where $\gamma_{0}$ is the initial energy intensity and $r_{\gamma }$ is the rate at which the energy intensity declines over time. Energy is composed of both carbon-based sources (fossil fuel) and carbon-free sources (e.g., solar or wind). We denote by $m_{t}$ the endogenous share of carbon-free sources in the energy mix and by $a_{t}$ the endogenous fraction of emissions that is captured and stored using CCS and other sequestration technologies at time *t*. We suppose that energy is measured in Giga tonnes of carbon (GtC) or its equivalent. Hence, residual carbon emissions entering the atmosphere from aggregate production at time *t* amount to $(1 - a_{t} )(1 - m_{t} )\gamma_{0} e^{{ - r_{\gamma } t}} Y_{t} .$

The cost of the energy mix rises with the share of carbon-free renewable energies $$ m_{t} . $$ We suppose that the cost of one unit of energy declines at the relative rate of technical progress in using renewable energy rather than fossil fuel, denoted by $$ r_{R} , $$ thus capturing the potential for future cost reductions as carbon-free technologies mature. We let this cost be $$ m_{t} H_{0} + \theta_{m}^{ - 1} m_{t}^{{\theta_{m} }} e^{{ - r_{R} t}} H_{1} $$ with $$ \theta_{m} > 1, \, H_{0} \ge 0 $$ and $$ H_{1} > 0. $$ Similarly, we suppose that the cost of sequestrating one unit of emissions is $$ \theta_{a}^{ - 1} a_{t}^{{\theta_{a} }} e^{{ - r_{A} t}} A_{1} $$ with $$ \theta_{a} > 1 $$ and $$ A_{1} > 0, $$ where the relative rate of technical progress in sequestration is denoted by $$ r_{A} $$ and captures the potential for future cost reductions as sequestration technologies mature. We let the cost of generating 1 GtC of fossil fuel be $$ G(t) = G_{0} e^{{ - r_{F} t}} , $$ where $$ G_{0} \ge 0 $$ denotes the initial cost and $$ r_{F} $$ is the rate of technical progress in producing fossil fuel (e.g., due to the invention of horizontal drilling in fracking).^5^ Our formulation is general and allows us to disentangle the dynamics of energy use per fuel type and energy efficiency.

We denote the price of carbon emissions by $$ P_{t} , $$ so that the total costs of the energy mix per unit of output are $$ Z_{t} \equiv \big[ m_{t} H_{0} + \theta_{m}^{ - 1} m_{t}^{{\theta_{m} }} e^{{ - r_{R} t}} H_{1} + \theta_{a}^{ - 1} a_{t}^{{\theta_{a} }} e^{{ - r_{A} t}} A_{1} (1 - m_{t} ) + G_{0} e^{{ - r_{F} t}} (1 - m_{t} ) + P_{t} (1 - a_{t} )(1 - m_{t} ) \big]\gamma_{0} e^{{ - r_{\gamma } t}} . $$ Minimising this cost we get the upward-sloping schedules for the proportion of the energy mix that consists of renewable energy (also known as the mitigation rate) $$ m_{t} $$ and the share of emissions that is sequestrated (also known as the abatement rate) $$ a_{t} : $$

1 $$ m_{t} = \left( {\frac{{G_{0} e^{{ - r_{F} t}} + \frac{1}{{\theta_{a} }}a_{t}^{{\theta_{a} }} e^{{ - r_{A} t}} A_{1} + (1 - a_{t} )P_{t} - H_{0} }}{{H_{1} e^{{ - r_{R} t}} }}} \right)^{{\varepsilon_{m} }} ,\quad 0 \le m_{t} \le 1, $$

2 $$ a_{t} = \left( {e^{{r_{A} t}} P_{t} /A_{1} } \right)^{{\varepsilon_{a} }} ,\quad 0 \le a_{t} \le 1, $$

where $$ \varepsilon_{i} = 1/(\theta_{i} - 1) > 0 $$ for *i* = *m,* *a* denote price elasticities.^6^ A higher carbon price $$ P_{t} $$ thus leads to both more substitution of renewable energy in the energy mix and to more sequestration of carbon emissions. More technical progress in renewable energies (higher $$ r_{R} $$) leads to a faster substitution of renewable energies for fossil fuel but does not affect sequestration. The rate of sequestration is only affected by its own technology parameters and increases as its cost falls (higher $$ r_{A} $$ and lower *A*_1_). Higher cost of fossil fuel and lower cost of renewable energies (higher $$ G_{0} $$ and lower $$ H_{0} $$ and $$ H_{1} ) $$ boost the share of energy mix that consists of carbon-free energies. Equation (1) imply that $$ m_{t} = m(t,P_{t} ) $$ and $$ a_{t} = a(t,P_{t} ), $$ and thus we can express minimal unit cost as $$ Z_{t} = Z(t,P_{t} ). $$ The share of carbon-free sources in the energy mix, the fraction of emissions that are sequestrated and the minimal unit energy cost thus depend on the carbon price and time (via the various rates of technical progress). In the absence of carbon pricing, no emissions are sequestered (*a*_t_ = 0) while renewable energies are still utilised to the point where their marginal cost equals that of fossil energy, reflecting current economic circumstances. In pushing up the cost of polluting energy sources, carbon pricing increases the share of renewables in energy generation and makes sequestration profitable.

The optimal pricing of carbon depends on the severity and duration of climate damage caused by one unit of carbon. We assume that, once carbon is emitted into the atmosphere, it evolves according to a two-box carbon cycle. The stock of atmospheric carbon $E_{t} \equiv E_{t}^{P} + E_{t}^{T}$ consists of a permanent part, which retains a share $0 < \beta_{0} < 1$ of carbon emissions. A transient part of atmospheric carbon, which retains a share $1 - \beta_{0}$ of carbon emissions, decays at the rate $\beta_{1} > 0.$ We suppose that there is an average lag *Tlag* before global mean temperature responds to an increase in the stock of atmospheric carbon. We capture this by letting the aggregate flow damage from global warming per unit of output be given by $d\,\tilde{E}_{t} ,$ where $\tilde{E}_{t}$ denotes the delayed carbon stock (i.e., after temperature has responded to changes in the atmospheric carbon). We can thus summarise our model of the dynamics of atmospheric carbon and temperature by

3 $$ \begin{aligned} \dot{E}_{t}^{P} & = \beta_{0} \left[ {1 - a(t,P_{t} )} \right]\left[ {1 - m(t,P_{t} )} \right]\gamma_{0} e^{{ - r_{\gamma } t}} Y_{t} , \\ \dot{E}_{t}^{T} & = (1 - \beta_{0} )\left[ {1 - a(t,P_{t} )} \right]\left[ {1 - m(t,P_{t} )} \right]\gamma_{0} e^{{ - r_{\gamma } t}} Y_{t} - \beta_{1} E_{t}^{T} , \\ \dot{\tilde{E}}_{t} & = (E_{t}^{P} + E_{t}^{T} - \tilde{E}_{t} )/Tlag. \\ \end{aligned} $$

Aggregate global consumption $C_{t}$ is what is left of aggregate global production after subtracting global warming damages and energy costs. If the revenue from carbon taxes (or from selling carbon emission permits) are rebated to the private sector, it is

4 $$ C_{t} = \left[ {1 - d\,\tilde{E}_{t} - Z(t,P_{t} ) + P_{t} \;\partial Z(t,P_{t} )/\partial P_{t} } \right]Y_{t} {\text{ as }}\partial Z(t,P_{t} )/\partial P_{t} = (1 - a_{t} (1 - m_{t} )\gamma_{0} e^{{ - r_{\gamma } t}} Y_{t} . $$

Climate policy maximises global welfare corresponding to the present discount value of utilities derived from the stream of present and future consumption levels,

5 $$ \varOmega \equiv \int_{0}^{\infty } {U(C_{t} )e^{ - RTI \times t} dt} {\text{ with }}U(C_{t} ) = \frac{{C_{t}^{1 - IIA} }}{1 - IIA}, $$

subject to the dynamics of the climate system (3), where *RTI* > 0 denotes the constant rate of time impatience and the utility function is iso-elastic with a constant coefficient of relative intergenerational inequality aversion, *IIA*. The *IIA* captures how little current generations are prepared to sacrifice current consumption to limit future global warming.^7^ Upon substitution of aggregate consumption from (3) and $Y_{t} = Y_{0} e^{ - gt}$ for trend aggregate world production, we get

5′ $$ \varOmega = \int_{0}^{\infty } {\left( {\frac{{\left[ {1 - d\,\tilde{E}_{t} - Z(t,P_{t} )} \right]Y_{0} }}{1 - IIA}} \right)^{IIA} e^{ - Rt} dt = } {\text{ with }}R \equiv RTI + IIA \times g, $$

where *R* denotes the (long-run) social discount rate (and corresponds to the one from the Keynes-Ramsey rule). The social discount rate is high if the rate of time impatience is high, future generations are richer than current ones (provided *IIA* > 1), and intergenerational inequality aversion is high (provided $g > 0$). The choice of the social discount rate has been subject to much debate. We have here a constant social discount rate, but will generalise our findings to non-constant discount rates in Sect. 6 where we combine relatively high short run discount rates suggested by Nordhaus (2008) with near-zero rates for the *RTI* as argued in the Stern Review.

Output grows at constant trend rate of growth *g*. What matters for optimal (climate) policy is the social discount rate corrected for growth denoted by

6 $$ SDR = R - g = RTI + (IIA - 1) \times g. $$

This growth-corrected discount rate takes into account the trade-off between greater material wealth when deciding how much climate mitigation to do. If intergenerational inequality aversion is high (*IIA* > 1), higher income growth pushes up the *SDR* and future damages are taken into account (relatively) less. With logarithmic utility (*IIA* = 1), the *SDR* is simply the *RTI*. When intergenerational inequality aversion is low (*IIA* < 1), current generations are willing to sacrifice their own consumption even as future generations get richer.

## Optimal Policies for Making the Energy Mix Carbon-Free

We can now conduct the cost-benefit analysis of choosing between fossil and renewable energy sources and the amount of emissions to be sequestered, having defined preferences, endowments, and technology. The following result presents our simple rules for the optimal carbon price, $$ P_{t} , $$ the optimal share of carbon-free sources in the energy mix, $$ m_{t} $$ (the mitigation rate), and the fraction of emissions that are sequestrated, $$ a_{t} $$ (the abatement rate) for our back-on-the-envelope IAM.

**Result 1:***The optimal carbon price is*

7 $$ P_{t} \cong \tau Y_{0} e^{gt} {\text{ with }}\tau = \left( {\frac{{\beta_{0} }}{SDR} + \frac{{1 - \beta_{0} }}{{SDR + \beta_{1} }}} \right)\left( {\frac{1}{1 + SDR\, \times Tlag}} \right)d, $$

*where the growth*-*corrected social discount rate SDR is* (6)*. Given* (7)*, the fraction of fossil fuel use that is abated and the share of renewable energies in total energy follow from* (1) *and* (2).

### *Proof*

see “Appendix 1”.

Expression (1) for our simple rule for the optimal price of carbon does not depend on the fossil fuel intensity of the economy, because along the balanced growth path the consumption share is fixed. The optimal carbon price is proportional to GDP and hence grows at rate *g*. The optimal carbon price is depressed by the lag between changes in temperature and in the stock of atmospheric carbon (Rezai and van der Ploeg 2016; van den Bijgaart et al. 2016). If the temperature lag is absent, (7) boils down to the simple rule derived in Golosov et al. (2014).^8^ The carbon price also depends on other *geophysical* factors. It increases in the share of emissions that stay permanently in the atmosphere (higher $\beta_{0} )$ and increases if the rate of decay of atmospheric carbon drops (lower $\beta_{1}$). The latter might occur if global warming has depressed the absorption capacity of the oceans and other carbon sinks. The *ethical* drivers of the carbon price can be seen from the *SDR*. If society is relatively impatient (high *RTI*) and shows little willingness to sacrifice current consumption to curb future global warming (high *IIA*), the *SDR* is high and thus carbon pricing is unambitious. Finally, the *economic* drivers of the carbon price are twofold. Higher economic costs of global warming resulting from a higher flow damage coefficient (higher *d*) or higher current GDP give rise to a higher price of carbon. With a higher trend rate of economic growth, future damages (being proportional to future GDP) will be higher and thus the present discounted value of these damages and the optimal carbon price will be higher too. Furthermore, if the rate of economic growth is high and thus future generations are relatively richer than the current generation, there is less willingness among the current generation to undertake ambitious climate policies (high $IIA \times g$ and thus a high *SDR* from (6)). This latter effect is captured by the negative effect of *g* on the growth-corrected *SDR* which dominates if growth *IIA* > 1.

These geophysical, ethical, and economic drivers of the optimal carbon price are also the drivers of the fraction of the energy mix that is clean and the fraction of carbon emissions that are sequestrated as these increase in the carbon price can be seen from (1) and (2), respectively. As discussed in Sect. 2, (1) and (2) also indicate that substitution for renewable energies and sequestration also increase if their marginal costs fall due to technical progress.

## Policy Regimes, Transition Times and Carbon Budgets

The emissions of carbon ends either by ongoing substitution of all fossil fuel for renewable energies or by full sequestration (i.e., when either $m_{t}$ or $a_{t}$ reaches 100%). Depending on which one stops the fossil era, we can identify the corresponding transition times and carbon budgets. For example, if the cost of using carbon-based energy (including the carbon tax) is greater than the cost of the carbon-free alternative, i.e.$G_{0} + \tau Y_{0} > H_{0} + H_{1} ,$ full mitigation is optimal from the start and $m_{t} = 1, \, \forall t \ge 0.$ No sequestration is necessary and the carbon budget and transition time are irrelevant. In fact, we suppose the more realistic case where carbon-free technologies are not competitive today or in the near future, i.e.,$G_{0} + \tau Y_{0} < H_{0} + H_{1} .$ This implies positive emissions with $m_{0} < 1$ and *m*_*t*_ rising monotonically over time, given that renewable energy becomes competitive over time relative to their carbon-based alternatives. In this scenario it is optimal to start with a phase where fossil fuel is used alongside renewable energies. If $A_{1} < \tau \,Y_{0} ,$ only part of these fossil fuel emissions are abated initially. In this case two regimes, with partial and complete sequestration of carbon emissions, are possible, before renewables take over fully in the third regime.

We first focus on the regime with partial sequestration, so at the time of transition to the carbon-free era, *T*, all energy consists of renewables, i.e. $m_{t} = 1$ for all *t* ≥ *T*, and not all emissions from burning fossil fuel are fully sequestrated yet, i.e., $a_{t} < 1$ for all *t* < *T*. There is no need for sequestration in the carbon-free era, so that $a_{t} = 0$ for all *t* ≥ *T*. The following result summarises such a regime with partial sequestration.

**Result 2 (partial sequestration):***If fossil fuel is completely removed from the energy mix before all emissions are fully sequestrated, i.e. m*_*t*_ = *1 for t* *≥* *T and a*_*t*_ < *1 for t* < *T, the optimal carbon price, the share of renewable energies in total energy, and the fraction of carbon emissions that are sequestrated follow from* (7), (1) *and* (2)*, respectively. Transition to the carbon*-*free era occurs once the cost of carbon*-*based energy, including the carbon price, has risen to just that of renewable energies or, equivalent, when m*_*T*_ = *1 has reached for some T. The optimal carbon budget corresponds to cumulative carbon emissions,*$B = \int_{0}^{T} {(1 - a_{t} )(1 - m_{t} )\gamma_{0} Y_{0} e^{{(g - r_{\gamma } )t}} dt} .$

 “Appendix 1” contains the formal statement and derivation of Result 3.^9^

The relevant arbitrage conditions for a regime where full mitigation occurs before full sequestration are $m_{T} = \big[ \big( G_{0} e^{{ - r_{F} T}} + \theta_{a}^{ - 1} a_{T}^{{\theta_{a} }} e^{{ - r_{A} T}} A_{1} + (1 - a_{T} )P_{T} - H_{0} \big)/\left( {H_{1} e^{{ - r_{R} T}} } \right) \big]^{{\varepsilon_{m} }} = 1$ and $a_{T} = \left( {e^{{r_{A} T}} P_{T} /A_{1} } \right)^{{\varepsilon_{a} }} < 1.$ Climate policy and technology jointly determine whether this regime occurs. We assume that there is sufficient technical change in renewable energy production, relative to cost reductions in dirty energy, so that the mitigation ratio rises with time until it reaches one and the switch to the carbon-free era takes place. Technological change and a carbon price rising at the rate of economic growth drive this transition. If there is no directed technical change whatsoever and no economic growth, the share of renewables in the energy mix is constant, $m_{t} = \left( {\frac{{G_{0} + P_{0} (1 - a) - H_{0} }}{{H_{1} }}} \right)^{{\varepsilon_{m} }} ,\;\forall t \ge 0,$ and the fraction of carbon emissions that is sequestrated is constant too, $a_{t} = \left( {\frac{{P_{0} }}{{A_{1} }}} \right)^{{\varepsilon_{a} }} ,\;\forall t \ge 0,$ so there will never be a switch to the carbon-free era. Hence, cumulative emissions rise forever and climate policy has become impotent. Carbon emissions cause global warming but the ensuing economic damages are evaluated as too low to warrant a more aggressive carbon tax.

The second regime occurs if substitution for renewable energies occurs at a too low pace relative to the pace at which sequestration takes place in which case it is optimal to sequestrate all carbon emissions at time *T′* before all fossil in the energy mix is fully replaced by renewables at time *T* > *T′,* with $T' = \frac{1}{{r_{A} + g}}\ln \left( {\frac{{A_{1} }}{{\tau Y_{0} }}} \right)$ and *T* from $G_{0} e^{{ - r_{F} T}} + \frac{1}{{\theta_{a} }}A_{1} e^{{ - r_{A} T}} = H_{0} + H_{1} e^{{ - r_{R} T}} .$ This regime is relevant if the cost of sequestration is low and technical change in sequestration is high, both relative to the cost of switching to renewable energies. For this regime there are three distinct potential phases: phase 1 where fossil fuel is partially sequestrated and used alongside renewable energies during the period 0 ≤ *t* < *T′*, phase 2 where fully sequestrated fossil fuel is used alongside renewable energies during the period *T′* ≤ *t* < *T*, and possibly a phase 3 where only renewable energies are used and sequestration is no longer necessary for the period *t* ≥ *T*. If technical change in the development of carbon-free alternatives is slow, phase 2 lasts longer and features a temporarily falling share of renewable in the energy mix, $m_{t} .$

**Result 3 (full sequestration):***If full sequestration takes place before all fossil fuel is removed from the energy mix, a*_*t*_ = *1, for T′* ≤ *t* < *T, the optimal carbon price and share of renewable energy in the energy mix are given by* (7) *and* (1)*. The fraction of emissions that are sequestrated in phase 1 follows from* (2) *before reaching the value of 1 in phase 2 at time T′*. *The transition time to phase 3, the carbon*-*free era, T, occurs once the cost of fully sequestrated carbon*-*based energies including the carbon price has risen to just the cost of renewable energies. The carbon budget, B, equals cumulative use in phase 1, from time 0 to T′.*

“Appendix 1” contains the formal statement and derivation of Result 3.^10^

Equations (1)–(2) with (6) and (7) define our back-of-the-envelope IAM. Climate policies in the form of substituting renewables in the energy mix and sequestration determine the transition time, *T*, at which the carbon era comes to an end, and the carbon budget *B*, by pricing carbon appropriately. From time *T* onwards, fossil fuel use is zero and all energy is carbon-free. Knowing the carbon budget, we can determine peak global warming (*PW*) using the relation *PW* = *Temp*_0_ + *TCRE* **×***B* (cf., Allen 2016), where *TCRE* is the transient climate response and *Temp*_0_ a constant.

Pricing carbon makes sequestration profitable (abatement rate *a*_t_ positive) and increases the share of mitigation, thereby shortening the transition time and the carbon budget. A higher carbon price (e.g. because of a higher damage coefficient for global warming *d* which pushes up the whole carbon price trajectory) increases the share of renewable energies in total energy, increases the fraction of carbon emissions that is abated, and brings forward the transition to the carbon-free era. This cuts the optimal carbon budget and peak global warming. Generally, the effectiveness of carbon pricing depends on technological possibilities and prospects. If the cost of renewable energy is falling fast, i.e. large *r*_*R*_, carbon pricing only adds little effect to the technologically driven transition to sustainability. Innovations like horizontal drilling which lead to the shale gas revolution can be captured as a big negative shock to *G*_0_. Although the carbon price is unaffected, the transition to the carbon free era is postponed as it is profitable to continue with fossil fuel for longer. Furthermore, the ratio of renewable energy in total energy drops instantaneously and as a consequence the optimal carbon budget and peak global warming are higher. A breakthrough in renewable energy production captured by a negative shock to *H*_0_ has the opposite effects. A strong enough breakthrough in sequestration technology also tilts the policy mix toward abatement away from mitigation, permitting a regime with 100% abatement (see Sect. 7.3). If technical change in renewables, *r*_*R*_, is strong compared with that in fossil fuel extraction, *r*_*F*_, and sequestration, *r*_*A*_, carbon-free technologies eventually gets cheap enough to replace fossil fuel *cum* sequestration, so that the transition time *T* is finite. If technical change in renewable energies is sufficiently rapid, sequestration only plays an important transitional role in the intermediate phase before the economy abandons fossil fuel altogether.

Without climate policy, i.e. *P*_*t*_ = 0, technological progress and cost-cutting in carbon-free technologies are still able to drive carbon emissions to zero. The introduction of a carbon price shortens this transition period. The carbon budget is small for a high and rapidly rising extraction cost of fossil fuel and social cost of carbon, and a low and rapidly falling cost of renewable energy and abatement.

## Optimal Climate Policies: Benchmark Calibration

Table 1 gives the ethical, economic and geophysical assumptions underlying the benchmark calibration of our back-of-the-envelope IAM. Unless stated otherwise, this follows the DICE and RICE models (Nordhaus 2010, 2015) for the ethical parameters and economic growth, cost and technological parameters, and baseline scenarios (see “Appendix 3” for more details). As far as the ethics is concerned, time impatience is 1.5% per year and the coefficient of relative intergenerational inequality aversion, *IIA*, is 1.45. Given a trend rate of economic growth of 2% per year, the Keynes-Ramey rule implies an interest rate of 4.4% per year and thus the growth-corrected social discount rate, *SDR*, is 2.4% per year.

**Table 1:** Benchmark calibration

*Ethical* Rate of time impatience for exponential discounting: *RTI* = 1.5% per year Intergenerational inequality aversion and risk aversion: *IIA* = 1.45 Growth-corrected social discount rate: *SDR* = 2.4% per year
*Economic* World economy: *GDP*_0_ = 73 T$, *g* = 2% per year Energy use per unit of world GDP: *γ* = 0.14 GtC/T$, *r*_*γ*_ = 0% per year Fossil fuel cost: *G*_0_ = 515 $/tC, *r*_*E*_ = − 0.1% per year Renewable energy cost: *H*_0_ = 515 $/tC, *H*_1_ = 1150 $/tC, *θ*_*m*_ = 2.8, *ε*_*m*_ = 0.55, *r*_*R*_ = 1.25% per year Sequestration (CCS) cost: *A*_1_ = 2936 $/tC, *θ*_*a*_ = 2 so *ε*_*a*_ = 1, *r*_*A*_ = 1.25% per year Flow damage as fraction of world GDP: *d* = 0.019 $/tC
*Geophysical* Coefficients permanent & transient box of carbon cycle: *β*_0_ = 0.2, *β*_1_ = 0.0023 Average lag between temperature/damages and carbon stock: *Tlag***=** 10 years Transient climate response to cumulative emissions: *TCRE* = 2 °C/TtC

For the economics, energy use is 0.14 Giga tons of carbon per trillion dollars of world GDP (initially $73T) amounting to 10 GtC of emissions. The initial cost of fossil fuel is 7% of GDP or $515/tC, and we assume cost rises at 0.1% per year to capture higher costs as less fossil fuel reserves remain. The unit cost of fossil fuel is constant (resulting in a constant energy share in the absence of climate policy) while the unit cost of renewable increases as their share in the energy mix rises. The corresponding price elasticity is 0.55 and the rate of technical progress in carbon-free energy is 1.25% per year. Sequestration is not captured explicitly in the DICE model. We assume that the cost of sequestration is initially quite high, namely 20% of GDP (or $2936/tC), and declines at the same rate of technical progress as renewables (at a rate of 1.25% per year). We set the cost of global warming at 1.9% of world GDP (measured in trillions of dollars) for every trillion ton of carbon.^11^

We adopt the geophysics from the model of Golosov et al. (2014) and assume that 20% of carbon emissions remain forever in the atmosphere and the remainder returns back to the surface of the oceans and the earth at a speed of 0.23% per year. We add a mean lag of 10 years between the rise in temperature and the change in the stock of atmospheric carbon. Following Allen (2016), we let the transient climate response to cumulative emissions be 2 °C per trillion tons of carbon.

Since the ethics and the costs and benefits of climate policies in the near and very distant future are open for debate and to a much lesser extent the geophysics too, the assumptions in Table 1 are to a certain extent subjective. Our framework, however, allows us to investigate the effects of changing these assumptions on optimal climate policies in a transparent, straightforward way (see Sects. 6–9).

**Fig. 1:**
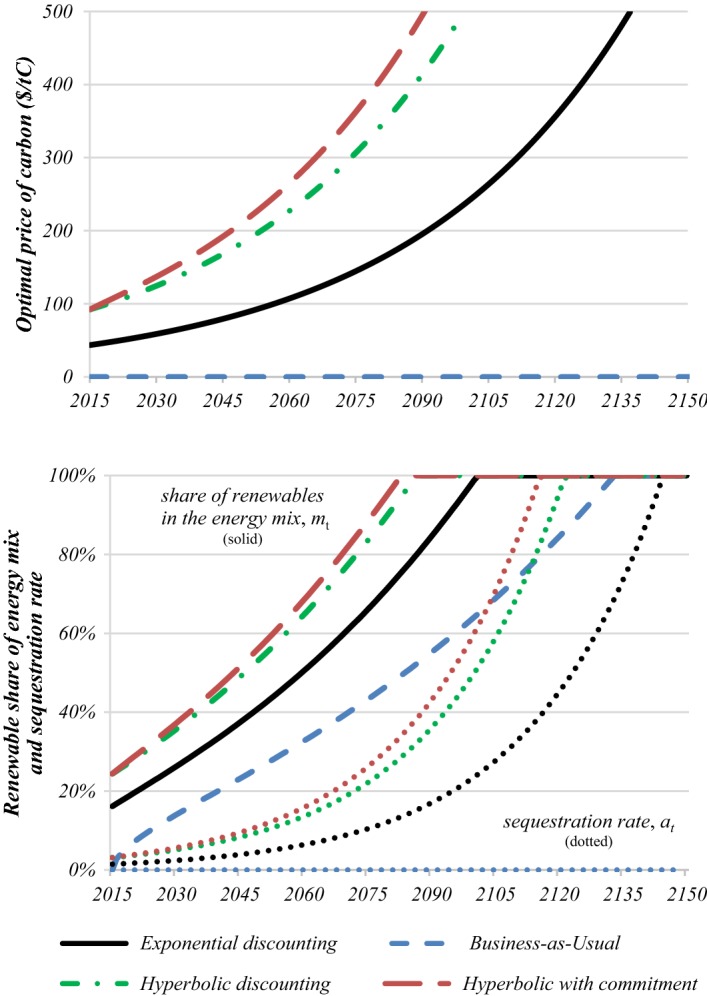
Optimal climate policy under hyperbolic discounting. *Key* Under hyperbolic discounting without commitment (green dashed-dotted lines) climate policy is more ambitious than under exponential discounting (black lines), where less weight is placed on future generations’ welfare. Even in the absence of a carbon price (blue short-dashed lines) fossil fuels are slowly phased out due to the advance of carbon-free technologies. Carbon prices can be compared to the less plausible case of hyperbolic discounting with pre-commitment (brown long-dashed lines). (Color figure online)

Given our benchmark calibration in Table 1, the solid black and short-dashed blue lines in Fig. 1 are the outcomes under the optimal climate policies and under business as usual (BAU) where the carbon price is zero, respectively. Our simple rule for the optimal carbon price starts at $44/tC (or $12/tCO_2_) and then grows in line with the trend rate of economic growth at 2% per year—see the top panel. The black solid line in the bottom panel shows that the mitigation rate starts at 16% and then rises to 100% in 86 years, growing on average at 2% per year. Pricing carbon leads to 1.5% of the remaining fossil fuel emissions being sequestrated initially (see the red dotted line in the bottom panel of Fig. 1). Following (2), sequestration increases at a progress-adjusted growth rate of 3.25% per year. By the end of the carbon ear, a total of 784 GtC have been emitted, inducing peak warming of 2.9 °C early in the next century due to the 10-year average lag in the climate system.

**Fig. 2:**
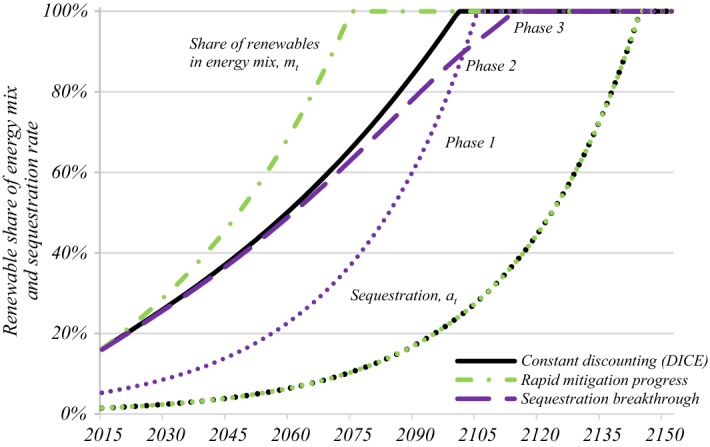
Technological drivers of climate policies. *Key* Technological improvements in renewable energies have a significant impact on the carbon budget and peak warming while reductions in the cost of sequestration mostly affect the composition of emission reduction, phasing in sequestration more slowly while completely switching over to renewables is delayed. (Color figure online)

Without a carbon price, cost reductions in the generation of renewable energy are the only drivers of the energy transition. Fossil fuels are used more and for longer, with *m*_*t*_ in the second panel of Fig. 2 rising slowly towards full decarbonisation in the next century. Without the carbon price stick, no sequestration efforts will be undertaken, increasing emissions further. If no additional policy measures are imposed (such as fuel standards, renewable subsidies, a moratorium on coal, etc.), BAU leads to cumulative emissions of 1778 GtC and peak warming of 4.9 °C. Positive mitigation levels under BAU are solely driven by the gradual improvements in the cost competitiveness of renewable energy. If the cost differential between dirty and clean inputs were to remain constant, i.e. $$ r_{R} = r_{F} = 0, $$ carbon-based technologies would be used indefinitely, i.e., *m*_*t*_ = 0, under BAU.

Despite its simplicity, our IAM compare well with the fully-fledged DICE-2013R model to which we have calibrated our model parameters. In the absence of population growth, DICE reports an initial carbon price of $48/tC and mitigation rate of 17%. The rates of growth of the carbon price and mitigation are, however, significantly slower and cumulative emissions higher due to DICE’s long temperature lag of more than 100 years. In our simulations we have also verified that the approximation of a constant consumption share, used in our simple rule (7), is reasonable for our chosen calibration. Along the policy paths shown in Fig. 1, this ratio varies between 93% and 90% over time, since energy and damages constitutes only a modest share of GDP along an optimal path where climate damages are limited.

## Ethics: Low Discount Rates for the Long Term and Affluence of Future Generations

Here we discuss the question of discounting, first in Sect. 6.1 an extension of the benchmark model to hyperbolic discounting and then briefly discuss ethical considerations in connection with intergenerational inequality aversion and wealth of future generations in Sect. 6.2.

### Hyperbolic Discounting Versus Exponential Discounting

The rate of time impatience, *RTI*, represents the weight placed on future generations’ welfare and crucially determines how ambitious climate policy is. Our welfare function (5) with exponential discounting implies that *RTI* is constant. Given a constant rate of trend economic growth, the growth-corrected social discount rate, *SDR*, is constant too (see Eq. (6)). A smaller *RTI* lowers the *SDR* and increases the carbon price (7) and thus makes climate policy makes more ambitious. Our purpose is to extend our analysis to *hyperbolic discounting*, which nests our base calibration with exponential discounting and constant *RTI* as a special case. Empirical and theoretical arguments support the declining long-term discount rates being lower than short-term discount rates (e.g., Arrow et al. 2013, 2014). The presence of risk or heterogeneous agents have been put forward as a compelling arguments for certainty-equivalent rates that decline with long time horizons (e.g., Weitzman 1994, 2001; Gollier and Zeckhauser 2005). Microeconomic studies on procrastination suggest that people tend to delay beneficial but hard actions (Laibson 1997).^12^ Our motivation for using generalised hyperbolic discounting is that it allows us to use a high short-run discount rate, $\rho ,$ which we associate with the more *market*-*based* rate of time preference, and a much lower or zero long-run discount rate, which we associate with an *ethical* rate of time preference.^13^

The general class of hyperbolic discounting has discounting function $$ D_{t} = \left( {1 + \iota t} \right)^{{ - \frac{\rho }{a}}} , $$$$ \iota \ne 0. $$ For *ι* → 0, this simplifies to exponential discounting,$$ D_{t} = e^{ - \rho t} , $$ which was used in our benchmark welfare function (5). With $$ \iota = \rho , $$ we get the case of hyperbolic discounting, $$ D_{t} = \left( {1 + \rho t} \right)^{ - 1} . $$ The instantaneous discount rate at time *t* is defined as $$ \delta_{t} \equiv - D_{t} '/D_{t} = \rho /(1 + \iota t) $$ and equals *ρ* at time zero and then declines to zero as time goes to infinity. With this type of discounting and in contrast to the benchmark case of exponential discounting, optimal (climate) policies are generally time inconsistent. Hence, if policy makers re-optimise at some future point of time and renege, they will choose different policies. We therefore distinguish between optimal climate policies with commitment and those without commitment.

**Result 4:** *With commitment and generalised hyperbolic discounting, the optimal carbon price is*

8 $$ \begin{aligned} P_{t}^{commitment} & = d\,Y_{0} \int_{t}^{\infty } {\left( {\frac{1}{1 + \iota \,t}} \right)^{{\frac{\rho }{\iota }}} e^{ - (IIA - 1)gt} \left[ {\beta_{0} + (1 - \beta_{0} )e^{{ - \beta_{1} t}} } \right]dt} \\ & = dY_{t} \,\left( {\iota^{ - 1} + t} \right)e^{{g(IIA - 1)(\iota^{ - 1} + t)}} \\ & \quad \times \left\{ {\beta_{0} E_{{^{\rho /a} }} \left( {g(IIA - 1)(\iota^{ - 1} + t)} \right)} + \frac{{1 - \beta_{0} }}{{1 - \beta_{1} \times Tlag}}e^{{\beta_{1} \left( {\iota^{ - 1} + t} \right)}} E_{{^{\rho /a} }} \left( {\left[ {g(IIA - 1) + \beta_{1} } \right](\iota^{ - 1} + t)} \right) \right. \\ & \quad - \left. {\left( {\beta_{0} + \frac{{1 - \beta_{0} }}{{1 - \beta_{1} \times Tlag}}} \right)e^{{Tlag^{ - 1} \left( {\iota^{ - 1} + t} \right)}} E_{{^{\rho /a} }} \left( {\left[ {g(IIA - 1) + Tlag^{ - 1} } \right](\iota^{ - 1} + t)} \right)} \right\}, \\ \end{aligned} $$

*where* $E_{n} (x) \equiv \int_{1}^{\infty } {\frac{{e^{ - x\,s} }}{{s^{n} }}ds}$ *is the generalised exponential integral function.*

#### *Proof*

See “Appendix 2”.

The initial optimal carbon price under hyperbolic discounting (8) is *higher and rises at a faster rate* than the price under exponential discounting (7), since the discount rate falls with longer time horizons. The optimal carbon price (8) assumes commitment to an announced time path of future carbon prices. If the policy makers renege on predecessors’ plans and re-optimise at some future date, the carbon price is lowered again (due to the relatively high discount rate for short horizons) and rises monotonically as time progresses. In equilibrium, the carbon price is recalculated in each period and current policymakers take this into account when announcing their policies.

**Result 5:** *The optimal carbon price under generalised hyperbolic discounting when policy makers cannot commit to announced future time paths of carbon prices is*

8′ $$ P_{t}^{no - commitment} = (Y_{t} /Y_{0} )P_{0}^{commitment} . $$

#### *Proof*

“Appendix 2” shows that this corresponds to the feedback Nash equilibrium, which is time consistent by construction and relevant when commitment is not feasible.

We thus see that the optimal carbon price without commitment (8′) follows a lower trajectory than the carbon price with commitment (8) as discount rates are reset to their initial, higher value in each period whereas they are allowed to decline if policy makers can commit. As a result, carbon prices grow at a slower pace, namely at the rate of trend economic growth.

To illustrate how the assumption of generalised hyperbolic discounting affects climate policy, we calibrate the one-year discount rate for appraisal today to the one used by Nordhaus (2015), i.e. *δ*_0_ = 1.5% per year, and the one-year discount rate for in one century ahead to the one used by the Stern Review, i.e. *δ*_100_ = 0.1%/year. From $\delta_{t} = \rho /(1 + \iota \,t),$ this gives $\delta_{0} = \rho =$  1.5% and *ι*= [*ρ/δ*_100_ – 1]/100 = 0.14% per year. The discount rate is thus initially equal to the benchmark exponential rate but falls to 0.1%/year for a century ahead. Figure 1 and Table 2 report results for the case of generalised discounting and how they compare with the benchmark case of exponential discounting.

**Table 2:** Climate policy if future is discounted less heavily at longer horizons

	Carbon price *P*_*0*_	Sequestration *a*_*0*_ (%)	Mitigation *m*_*0*_ (%)	Carbon budget *B* (GtC)	End fossil era (years)	Peak warming (°C)
Exponential discounting (DICE)	44 $/tC	1.5	16.1	784	86	2.9
Hyperbolic discounting (no commitment)	92 $/tC	3.1	24.4	488	72	2.3
Hyperbolic discounting (with commitment)	92 $/tC	3.1	24.4	436	68	2.2
Business as usual	0 $/tC	0	0	1778	118	4.9
DICE	48 $/tC	–	17	1171	110	3.3

*Key* With exponential discounting there is a constant discount rate of 1.5% per year. Hyperbolic discounting starts with the same initial discount rate which then drops off over time to 0.1% per year in a century’s time. This leads to a much more ambitious climate policy with higher carbon taxes, higher sequestration and mitigation rates, lower carbon budgets and a quicker end of the fossil era. As a result, peak warming is less than with exponential discounting and much less than under business as usual. If commitment to future climate policies is feasible, carbon is initially taxed the same but then it grows at a faster rate so that climate policy is more ambitious. The value of commitment is small as it lowers the carbon budget by mere 52 GtC and peak warming by 0.1 °C. Under business as usual no carbon price is imposed and relative cost advances in renewable energy are the sole driver of decarbonisation. Here, the carbon era ends in the 22nd century with an excessive carbon budget and extreme levels of warming

The long-dashed red lines in Fig. 1 indicates the outcome under hyperbolic discounting if there is no commitment to announced future climate policies. The initial carbon price is much higher, $92/tC instead of $44/tC, but still rises in line with world GDP at the trend rate of economic growth of 2% per year. If policymakers can commit future policymakers to announced plans, indicated by the long-dashed grey lines in Fig. 1, the carbon price still starts at $92/tC, but rises initially more steeply at a rate 3.3% per year which then tapers off to a rate of 2% per year as the effect of declining discount rates fades. The declining discount rate thus makes climate policy more ambitious and especially so if policymakers can commit. If they renege and carbon prices are re-optimised after say 10 years, the carbon tax would be marked down by 8% and its growth rate reset to 3.3%. As a comparison, merely reducing the discount rate and sticking to exponential discounting lowers the initial carbon price, *P*_0_, but leaves the growth rate of the carbon price unchanged.

Hyperbolic discounting without commitment doubles the initial carbon price. This boosts the share of renewables in the energy mix by half to 24% and doubles sequestration rates to 3%. The start of carbon-free era is brought forward to the second half of this century, the carbon budget brought down to 488 GtC, and global mean temperature limited to 2.3 °C. If policymakers were to commit their future selves to their announcements about future climate policy, emissions and global warming would be reduced further to 436 GtC and 2.2 °C due the faster rising carbon price. Further comparisons of outcomes under hyperbolic discounting with and without commitment, exponential discounting and BAU are presented in Table 2.

Under the hyperbolic discounting case without commitment, the discount rate declined from 1% to 0.1% per year. The top part of Table 3 indicates that setting the constant, exponential discount rate *RTI* to this lower limit throughout gives even more weight to future generations and makes climate policy more ambitious. The initial rate increases to $108/tC, raising abatement and mitigation efforts significantly, limiting carbon emissions to 433 GtC and temperature increases to 2.2 °C.

**Table 3:** Ethic, economic, technological and geophysical drivers of optimal climate policies

	Carbon price *P*_*0*_	Sequestration *a*_*0*_ (%)	Mitigation *m*_*0*_ (%)	Carbon budget *B* (GtC)	Peak warming *PW* (°C)
Constant discounting (DICE)	44 $/tC	1.5	16.1	784	2.9
Lower discounting	108 $/tC	3.7	26.5	433	2.2
Higher inequality aversion	28 $/tC	1.0	12.7	966	3.2
Slower economic growth	55 $/tC	1.9	18.6	629	2.6
Higher damage	87 $/tC	3.0	23.6	509	2.3
Rapid mitigation progress	44 $/tC	1.5	20.2	388	2.1
Sequestration breakthrough	44 $/tC	5.3	19.9	595	2.5
More sophisticated carbon cycle	37 $/tC	1.3	14.7	854	3.0
Positive climate feedback	48 $/tC	1.5	16.4	754	2.8

### Intergenerational Inequality Aversion and Affluence of Future Generations

The top part of Table 3 also indicates that higher intergenerational inequality aversion within our benchmark model with (benchmark) exponential discounting makes climate policy more lacklustre, given continued economic growth. By increasing *IIA* from 1.45 to 2, the interest rate increases from 4.4% to 5.5% per year and consumption of current generations is judged as more valuable. Less stringent climate policy is enacted. The carbon price falls to $28/tC and the carbon budget and peak warming increase to 966 GtC and 3.2 °C, respectively.^14^ Note that the effect of *IIA* is stronger if the trend rate of economic growth is higher and future generations are relatively more affluent.

## Economics: Damages, Economic Growth, and Technical Progress

Table 3 also indicates that doubling the flow damage coefficient parameter (*d* = $0.038/tC) doubles the carbon price to $87/tC (or $24/tCO_2_) and implies a more ambitious climate policy. This nearly cuts the carbon budget in half to 509 GtC and limits the temperature rise to 2.3 °C.

### Effects of More Pessimism About Future Economic Growth

A slowdown in global economic growth to *g* = 1% per year makes future generations less affluent relative to current generations and thus makes current generations more willing to make sacrifices to curb future global warming as is evident from the decrease in the *SDR* especially if the *IIA* is high. The initial carbon price increases to $55/tC. There is an offsetting effect since a lower growth rate of the economy also means a lower growth rate of damages from global warming, which pushes down the *SDR* and depresses carbon pricing. The former effect dominates, since our benchmark calibration has *IIA* > 1. A lower rate of economic growth also reduces the growth rate of the carbon price. Table 3 shows that for our benchmark calibration the initial increase outweighs the slower growth of the optimal carbon price. As a result, carbon emissions and global warming are curbed by 155 GtC or 0.3 °C.

### Optimistic Scenarios for Technical Progress in Renewables Production and Sequestration

The DICE calibration used in our benchmark calibration is arguably too pessimistic about the potential of carbon-free technologies. Figure 2 and the bottom part of Table 3 therefore show the effects of doubling the speed of technological progress in carbon-free technologies (*r*_*R*_ = 2.5% per year) and of a breakthrough in sequestration technology, lowering the initial cost of full sequestration to that of renewable energies (*A*_1_ = 822 $/tC). Note from Result 1 that these cost variations leave the carbon price unchanged, but do affect the deployment of renewables and sequestration technologies and thereby the carbon budget and peak warming.

Doubling the speed in renewable innovation, *r*_*R*_, ramps up the adoption rate of renewables in the energy mix which reaches complete decarbonisation 16 years earlier than under the DICE-calibrated baseline. The carbon budget is reduced to 315 GtC and peak warming curbed to 2.2 °C. A technological breakthrough in sequestration, lowering the cost of decarbonisation under complete sequestration to that of full decarbonisation, has little effect on the statistics reported in Table 3. The cost reduction in sequestration relative to renewables lowers initial substitution efforts in the energy mix slightly while tripling sequestration. The cheap availability of sequestration leads to less emissions left unabated but pushes back the carbon-free era by over a decade. The carbon budget falls by 114 GtC and peak warming to 2.6 °C.

Given a strategy to price carbon, energy mix mitigation and sequestration policies can take on the two regimes discussed in Sect. 4. While regime I with partial sequestration dominates in most simulations, regime II with full sequestration occurs under the sequestration breakthrough scenario. After a long phase 1 with partial sequestration, full sequestration (phase 2) occurs briefly at the end of century before all fossil fuel is removed from the energy mix shortly after. Allen (2016) assumes that, while getting rid of all fossil fuel is never cost-effective, the share of renewables in the energy mix rises steadily with temperature over time and sequestrated emissions grow exponentially with sequestration continuing indefinitely. Our framework demonstrates important interactions between both policy instruments: once all emissions are sequestrated in phase 2, the replacement of fossil fuel by renewables stalls and rises more slowly as the pressure to limit climate change has been alleviated and relative cost considerations are the only determinants of the optimal policy mix between sequestration and substitution. In general, calibrated simulations show that bringing more renewable in the energy mix is the most important lever to avoid climate change with sequestration lowering transitions during the transition to the carbon-free era.

## Geophysics: Worsening of Absorptive Capacity of the Oceans with Global Warming

Our benchmark model of Sects. 2–5 has a simplified 2-box model of carbon dynamics and 1-box model of temperature dynamics. Typically, the geophysics is modelled in a more sophisticated way. If we have a *K*-box model for the carbon cycle, we have

3′ $$ \begin{aligned} \dot{E}_{t}^{P} & = \beta_{0} \left[ {1 - a(t,P_{t} )} \right]\left[ {1 - m(t,P_{t} )} \right]\gamma_{0} e^{{ - r_{\gamma } t}} Y_{t} , \\ \dot{E}_{t}^{Tk} & = \beta_{2k} (1 - \beta_{0} )\left[ {1 - a(t,P_{t} )} \right]\left[ {1 - m(t,P_{t} )} \right]\gamma_{0} e^{{ - r_{\gamma } t}} Y_{t} - \beta_{1k} E_{t}^{Tk} , \\ &\sum\nolimits_{k = 1}^{K - 1} {\beta_{2k} = 1,\;\beta_{2k} \ge 0,k = 1,..K - 1,} \\ \dot{\tilde{E}}_{t} & = \left( {E_{t}^{P} + (\sum\nolimits_{k = 1}^{K - 1} {E_{t}^{Tk} } ) - \tilde{E}_{t} } \right)/Tlag. \\ \end{aligned} $$

It is straightforward to demonstrate that the optimal carbon price in Result 1 then generalises to

7′ $$ P_{t} \cong \tau Y_{0} e^{gt} {\text{ with }}\tau = \left( {\frac{{\beta_{0} }}{SDR} + \left( {\sum\nolimits_{k = 0}^{K} {\frac{{\beta_{2k} (1 - \beta_{0} )}}{{SDR + \beta_{1k} }}} } \right)} \right)\left( {\frac{1}{1 + SDR\, \times Tlag}} \right)d, $$

For example, the IPCC uses the 4-box model for the carbon dynamics and 2-box model for the temperature dynamics put forward by Joos et al. (2013). Table 3 shows that the optimal climate policies are not much affected when we take the 4-box instead of the 2-box model.^15^ With the initial carbon price falling from $43.5 to $36.9/tC, the carbon era lasts slightly longer, the carbon budget increases by 70 GtC and peak warming by 0.1 °C. Following Millar et al. (2017), we also introduce positive feedback to the benchmark 2-box model in making the dissipation coefficients in (3) endogenous and hence representing the rate of absorption of carbon by the oceans as a decreasing function of global warming.^16^ Table 3 shows that the carbon price path and the substitution for renewable energies and sequestration rates are now slightly higher than either the linear 2-box or 4-box model for the carbon dynamics without positive feedback loops. This is because policy makers pursue a more ambitious climate policy to avoid unleashing unwelcome positive feedback loops.

## Politics: International Climate Deal Stalemates

So far we have assumed that countries in the world jointly determine policies addressing climate change as a global problem with a common global price of carbon. This presumes that lump-sum transfers flow from rich to poor countries to make sure that the internationally cooperative outcome can be sustained by all countries, even those that are poorer. After thirty years of international negotiations and despite some glimmers of hope at the Paris 2015 summit, the world is still far from an international deal on climate policy. One of the reasons for this is that rich countries do not want to compensate the poor countries enough for implementing global climate policy (Helm 2012). It is therefore of interest to compare the global first-best optimum presented in Result 1 with the outcomes when countries maximise their own welfare and do not cooperate with each other.^17^ One can distinguish two non-cooperative outcomes: a no-commitment outcome (or feedback Nash equilibrium) when countries cannot commit to future policies and condition their climate policies on the state of the economy (i.e., the stock of atmospheric carbon) and a commitment outcome (or open-loop Nash equilibrium) when each country can commit (van der Ploeg and de Zeeuw 1992). In our case, the two non-cooperative outcomes coincide as the only state variables are the permanent and transient stocks of carbon in the atmosphere and our rules for the optimal climate policies are independent of these.^18^

We attribute at each point in time country-specific flow damages from global warming to each country *i*, i.e., $$ d_{i} Y_{i} , $$ so that global flow damages sum to $$ \sum\nolimits_{i = 1}^{N} {d_{i} Y_{i} } = dY $$ where the weighted average of the flow damage coefficients is $$ d \equiv \sum\nolimits_{i = 1}^{N} {d_{i} Y_{i} } /Y. $$ In the non-cooperative case, countries only account for their country-specific damage when setting a price for carbon.

**Result 6**: *The optimal carbon price for the non*-*cooperative outcome is*

9 $$ \begin{aligned} P_{it} = \tau_{i} Y_{i0} e^{{g_{i} t}} \quad \quad with \, \tau_{i} &= \left( {\frac{{\beta_{0} }}{{SDR_{i} }} + \frac{{1 - \beta_{0} }}{{SDR_{i} + \beta_{1} }}} \right)\left( {\frac{1}{{1 + SDR_{i} \times Tlag}}} \right)d_{i} \, \hfill \\ & \qquad and \, SDR_{i} = RTI_{i} + (IIA_{i} - 1)g_{i} . \hfill \\ \end{aligned} $$

*where the fraction of renewable energies in the energy mix and the fraction of fossil fuel that is sequestrated are given by (1) and (2), respectively.*

Due to international free-rider problems, non-cooperative carbon prices are a factor *N* lower than under international policy cooperation if countries are equal in size and other respects. Poorer countries tend to suffer more from global warming (high *d*_*i*_) but still their desired carbon price is typically lower due to their GDP being much lower. To the extent that poor countries are catching up and have higher growth rates, their desired carbon prices will be lower still (provided their *IIA* exceeds unity). The transition to the carbon-free era will take longer under non-cooperation, especially if GDP levels and growth rates are distributed unevenly.^19^ Given lower carbon prices mitigation and sequestration rates are also lower without an international climate deal, especially in poorer countries.

To illustrate, we use the regional damage coefficients and initial regional GDP levels (from the World Bank data base) presented in Table 4. This disaggregation follows the RICE-2010 IAM (Nordhaus 2010) and uses the ensuing regional flow damage coefficients (Hassler and Krusell 2012). In RICE a 2.5% increase in global mean temperature causes an output-weighted loss of 1.5%, but in Africa and Europe this figure is 2.61 and 1.89 times as much, respectively, whilst in China and the US these ratios are only 0.15 and 0.3, respectively. The damage coefficients for global warming are thus high in Africa and in Europe compared to the US and especially China. Looking at regional flow damages per ton of carbon in the atmosphere (the third row in Table 4), we see that they are highest in Europa (due to both a high GDP and a high damage coefficient) and the rest of the world (ROW) but lowest in Africa (due to a low GDP and despite a high damage coefficient) and China (due its relatively low damage coefficient). This suggests that Europe has a much stronger interest in an international climate deal than Africa or China and also more the than the US. The choice of regions is naturally arbitrary but it serves to illustrate the biases in national climate policies when an international climate deal has not been achieved.

**Table 4:** Calibration of damages by world regions

*Regional damage flow coefficients (as multiples of global coefficient d*): *d*_*Africa*_ = 2.61 *d*, *d*_*Europe*_ = 1.89 *d*, *d*_*US*_ = 0.3 *d*, *d*_*China*_ = 0.15 *d, d*_*ROW*_ = 1.13 *d*
*Regional GDP levels for 2015* *GDP*_0*,Africa*_ = 2 T$, *GDP*_0*,Europe*_ = 16.8 T$, *GDP*_0*,US*_ = 18 T$*, GDP*_0*,China*_ = 10.8 T$, *GDP*_0,ROW_ = 25.7T$
*Regional flow damages per ton of carbon in the atmosphere (d*_*i*_ *GDP*_*i*_ *)* Africa $0.0992/tC, Europe $0.603/tC, US $0.103/tC, China $0.031/tC, ROW $0.552/tC

**Fig. 3:**
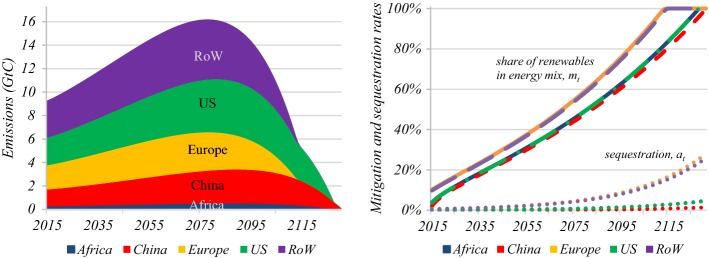
Non-cooperative regional climate policies. *Key* Failure to reach a global climate deal increases emissions. Each country tries to avoid its own damages but ignores those inflicted on others. Differences in exposure to climate change and income levels lead to varying degrees of ambition in climate policy. Europe with high levels of exposure and income decarbonises first; China with low exposure and income takes longest to drive emissions to zero. (Color figure online)

Figure 3 and Table 5 presents global emissions and regional climate policy when countries or regional blocks cooperate with each other and when they do not. We assume that cost and social welfare parameters are the same in all regions of the world.^20^ Due to international free-rider problems self-interested climate policy without an international agreement is significantly less ambitious than the first-best outcome discussed in the previous sections with lower shares of renewables in the energy mix and sequestration ratios, later transitions to full sequestration and a fully carbon-free energy mix, and consequently higher emissions in total for the global economy. The average carbon price without a climate deal starts at only $11/tC, only a quarter of what it would be under international policy cooperation. Global warming peaks thus at 4.0 °C instead of 2.9 °C and the carbon budget is 1352 GtC instead of 784 GtC. These poor outcomes under non-cooperation are likely to be even worse when countries in each regional bloc do not cooperate.

**Table 5:** Regional climate policy and global carbon budgets

Region	Carbon price *P*_*0*_	Abatement *a*_*0*_ (%)	Mitigation *m*_*0*_ (%)	Carbon budget *B* (GtC)
Africa	3.1 $/tC	0.1	3.8	44
China	1.0 $/tC	0.0	1.9	253
Europe	18.9 $/tC	0.6	10.2	257
US	3.2 $/tC	0.1	3.8	393
Rest of the World (RoW)	17.4 $/tC	0.6	9.7	405
Global cooperative	44 $/tC	1.5	16.1	784
Global non-cooperative	11 $/tC	0.4	7.1	1352
Business as usual	0 $/tC	0	0	1778

*Key* Regional climate policy is significantly less ambitious than under global policy coordination. Aggregating the regional policy responses (upper part of the table) to global averages (lower part), gives a carbon price of a quarter of the globally optimal, a carbon budget of 1352 GtC, and peak warming of 4.0 °C which compares favourably with 4.9 °C under business-as-usual but is far above the 2.9 °C under international policy coordination and cooperation

Without international transfers from rich to poor countries, the poorest regions in the global economy have little appetite to implement ambitious climate policies. This is why Africa and to a lesser extent China have relatively low carbon prices and thus relatively low abatement and mitigation ratios. The US has, of course, the highest level of GDP, but its optimal non-cooperative carbon tax is nevertheless very low as the damage coefficient for the US is relatively small. Due to a very low damage coefficient and a low level of GDP, China has the lowest price of carbon. Consequently, these two regions take the longest to reduce emissions to zero (see left panel in Fig. 3). Given their high initial carbon prices, Europe and the rest of the world are the quickest in phasing out fossil fuels. Due to the absence of regional differences in economic and technological growth rates, Africa and the US are following the same mitigation and abatement paths. A more disaggregated analysis would account for such differences which, due to lower technological capabilities in Africa than in the US, would lead to lower efforts to replace fossil fuel by renewables in Africa and strengthen ambition in the US.

## Conclusions

The central questions of climate policy are how much and how fast to replace fossil fuel with renewable energies in the energy mix; how much and how fast to sequestrate emissions from using fossil fuel; and how to set the initial and future prices of carbon both under non-cooperative and cooperative decision making in the global economy to achieve these goals. Much of the academic debate in climate change economics has focused on the difference of various estimates for the optimal carbon price, perhaps most prominently in the debate between Nordhaus and Stern about discount rates. We present a simple framework with which these questions can be meaningfully addressed without resorting to one of the large-scale numerical Integrated Assessment Models. Our back-of-the-envelope Integrated Assessment Model provides simple rules for the optimal price of carbon and for optimal share of renewables in the energy mix and sequestration policies and allows translation of these into climate objectives such as carbon budgets and peak warming. Our framework calculates the welfare-maximising carbon price but also pays attention to the dynamics of the technological capabilities available at different points of time. The carbon price and relative cost competitiveness determine the adoption and diffusion rate of carbon-free technologies. We see our analysis as complementary to more detailed, often numerical, simulations as we focus on the key drivers and ignore potential cross-interactions between capital accumulation and climate policy. Our aim was to develop a simple and easy-to-understand framework that brings together various aspects of climate change economics and can be used for teaching and for communication to policy makers to illustrate four key messages.

First, the optimal price of carbon and the ambition of climate policy are crucially driven by ethical considerations such as the discount rate and relative intergenerational inequality aversion on how to trade off the welfare of future and current generations. In a DICE-based calibration with a constant rate of time preference of 1.5% per year the current carbon price is $44/tC (or $12/tCO_2_) which increases to $146/tC if one adopts the discount rate of the Stern Review. We have shown how the standard framework with exponential discounting can be extended to allow for high discount rates at short horizons and much lower discount rates at long horizons by adopting a hyperbolic discounting approach. This hybrid case, which is our preferred estimate, respects the criticism of Nordhaus and Stern of each other’s approach and significantly boosts the optimal price of carbon to $92/tC (or $25/tCO_2_) and the speed at which fossil fuel is removed from the energy mix and emissions are sequestered to limit peak warming to 2.3 °C. Our framework takes into account that future policymakers might not want to respect the past climate pledges; our solution does not assume commitment. If future policymakers could commit to announcements about future policies, climate policy is more ambitious but time-inconsistent. The ability to commit, however, has minor implications for the carbon budget and peak warming.

Second, the qualitative nature of climate policy depends on how the economic and global warming costs of renewable and fossil energies and sequestration develop with time. If technical progress in renewable energy production is fast compared with that in developing sequestration technology, the economy replaces all fossil fuel by renewable energies (100% mitigation) before all fossil fuel is fully sequestrated. If technological progress in sequestration is relatively fast or if there is a breakthrough in sequestration technology, there will be an intermediate regime where all fossil fuel is fully sequestered before the economy finally transitions to using only renewable energies. During this intermediate phase of full sequestration, the urgency of climate policy recedes and the share of renewables in energy generation stalls. Due to current cost conditions and the ugly dynamics of NIMBY politics and running out of holes to put sequestrated carbon in, this second regime appears unlikely.

Third, as far as the geophysics is concerned, using a 4-box instead of a 2-box model of the carbon does not affect the optimal carbon price much. But allowing for the capacity of the oceans to absorb CO_2_ to diminish as oceans heat up, pushes up the optimal carbon price somewhat in order to avoid such positive feedback loops being set in motion.

Fourth, lack of international climate deals implies that carbon pricing and thus climate policies are nationally determined and lacklustre with the result that the necessary transition to the carbon-free era is much delayed and peak warming increases by an additional 1.1 °C. Part of the problem is failure of the rich countries to compensate the poor countries adequately for implementing an ambitious carbon price. This is why it is crucial to start with a club of countries who implement ambitious climate policies and generate mechanisms to get as many countries to join. A relatively low penalty trade tariff on countries outside the club of 5%, waived once they join, can lead to large and stable coalitions of countries and overcome free-riding in international climate policy (Nordhaus 2015).

It is straightforward to extend these back-of-the-envelope calculations to allow for more convex damages, stock-dependent fossil fuel extraction costs, sequestration costs that increase with the stock of sequestrated carbon, and learning by doing in renewables production (see “Appendix 5”). Since there is mounting evidence that climate policy shapes technological progress and research & development and that directed technical change and path dependence matters (e.g., Acemoglu et al. 2016; Aghion et al. 2016), it is important to allow for such endogenous feedbacks.

Our analysis solely considered climate-related damages to the economy without considering other non-climate implications of carbon-based processes. According to some estimates, such costs to health and well-being are of the same magnitude as the climate-related damages and might be important drivers of climate policy in certain regions (West et al. 2012; Ščasný, et al. 2015). E.g., while our regional analysis suggests that China should adopt few efforts to eliminate fossil fuel from the energy mix in the absence of a climate deal, non-climate co-benefits have been the key motivation for decarbonisation of industrial processes and fast ramping-up of renewable energy sources in this region. Our simple rules could be easily extended to include such additional costs of fossil fuels by adjusting the damage coefficients and thus the carbon price upwards.

Our simple framework may give too cautious answers as more convex global warming damages, damages to the growth rate of the economy and the risk of a cascade of catastrophic events which are more likely to occur at higher temperatures lead to a more aggressive climate policy (Dietz and Stern 2015; Rezai and van der Ploeg 2016; Lemoine and Traeger 2016; Cai et al. 2016), but can be extended to allow for such factors. If future climate policy turns out to be not ambitious enough and takes too long to materialise, there will be no other option than to attempt to curb negative carbon emissions via bio-energy with CCS, direct air capture or enhanced weathering as these forms of “negative emissions” may be needed to bridge the gap between cuts to meet global mitigation targets and current emissions trends (e.g., Fuss, et al. 2014).

Finally, as can be seen from Table 2, the usual DICE damages lead to peak global warming higher than 2 °C (unless one uses the very low discount rate of the Stern Review or the very low long-run discount rates that prevail under hyperbolic discounting with commitment). This contradicts the aims of the 2016 Paris Agreement to limit global warming to 2 °C and to aim to for peak warming of 1.5 °C. The Paris caps on peak global warming are, on the one hand, scientifically motivated as higher temperatures would lead to intolerably high risks of tipping points, and, on the other hand, politically motivated to keep small island states that are at risk of flooding aboard. One approach is to revise the damages from global warming upwards to ensure that peak global warming remains below 2 °C or 1.5 °C. The integrated assessment literature, in contrast, ignores damages from global warming altogether and minimises the present discounted value of costs subject to the constraint that peak warming cannot exceed 2 °C or 1.5 °C or alternatively that cumulative carbon emissions stay within the safe carbon budget corresponding to the cap on global warming. The resulting price of carbon must rise more rapidly to reflect that carbon gets scarcer as the carbon budget approaches exhaustion. In fact, the carbon price follows a Hotelling path and thus rises more rapidly at a rate equal to the rate of interest instead of the rate of economic growth (e.g., Nordhaus 1982; Tol 2013; Bauer et al. 2015).^21^ One way of integrating the welfare maximisation approach based on estimates of global warming and the cost minimisation approach based on a cap on global warming or cumulative emissions is to maximise welfare net of global warming subject to the cap on peak warming or cumulative emissions. This gives a cost-minimising price of carbon that is higher than under unconstrained welfare maximization and that grows at a rate somewhere in between the interest rate and the rate of economic growth (van der Ploeg 2018; Dietz and Venmans 2018).

## Appendix 1: Proof of Results 1–3

Global welfare is $$ \int_{0}^{\infty } {D_{t} U(C_{t} )dt} , $$ where $$ U(C_{t} ) = \frac{{C_{t}^{1 - IIA} }}{1 - IIA} $$ (for $$ IIA \ne 1 $$, $$ U(C_{t} ) = \ln (C_{t} ) $$ else) is time separable and has constant intergenerational inequality aversion and $$ D(t) $$ the discount rate at time *t* with $$ D'(t) \le 0. $$ Most economic analyses assume exponential discounting, i.e.$$ D_{t} = e^{ - \rho t} $$ with a constant rate of time impatience $$ RTI = \rho . $$ Using small letters to denote fractions of output before damages (e.g., $$ c_{t} \equiv C_{t} /Y_{t} $$), climate policy $$ \left\{ {a_{t} ,m_{t} } \right\}_{t = 0}^{\infty } $$ is chosen to maximise global welfare, $$ \int_{0}^{\infty } {\frac{{c_{t}^{1 - IIA} }}{1 - IIA}D_{t} e^{g(1 - IIA)t} dt} , $$ subject to the resource constraint $$ 1 - dE_{t} = c_{t} + $$$$ \left[ {\left( {G_{0} e^{{ - r_{F} t}} + \frac{1}{{\theta_{a} }}A_{1} e^{{ - r_{A} t}} a_{t}^{{\theta_{a} }} } \right)(1 - m_{t} ) + H_{0} m_{t} + \frac{1}{{\theta_{m} }}m_{t}^{{\theta_{m} }} H_{1} e^{{ - r_{R} t}} } \right]\gamma_{0} e^{{ - r_{\gamma } t}} . $$ The social consumption discount rate given by $$ CDR = RTI + IIA \times g $$ and the growth-corrected interest rate by $$ SDR = RTI + (IIA - 1)g = CDR - g. $$

The Hamiltonian for this problem with *r* equal to the *SDR* is

$$ \begin{aligned} {\rm H} & \equiv \frac{1}{1 - IIA}\left[ {1 - d\tilde{E}_{t} - \left( {H_{0} m_{t} + \frac{1}{{\theta_{m} }}m_{t}^{{\theta_{m} }} H_{1} e^{{ - r_{R} t}} } \right)\gamma_{0} e^{{ - r_{\gamma } t}} } \right. \\ & \left. {\quad - \left( {G_{0} e^{{ - r_{F} t}} + \frac{1}{{\theta_{a} }}A_{1} e^{{ - r_{A} t}} a_{t}^{{\theta_{a} }} } \right)(1 - m_{t} )\gamma_{0} e^{{ - r_{\gamma } t}} } \right]^{1 - IIA} + \tilde{\lambda }(E_{t}^{P} + E_{t}^{T} - \tilde{E}_{t} )/Tlag \\ & \quad { + }\,\lambda_{P} \beta_{0} (1 - a_{t} )(1 - m_{t} )\gamma_{0} e^{{ - r_{\gamma } t}} Y_{0} e^{gt} + \lambda_{T} \left( {(1 - \beta_{0} )(1 - a_{t} )(1 - m_{t} )\gamma_{0} e^{{ - r_{\gamma } t}} Y_{0} e^{gt} - \beta_{1} E_{t}^{T} } \right). \\ \end{aligned} $$

where $$ \lambda_{Pt} ,\;\lambda_{Tt} $$ and $$ \tilde{\lambda }_{t} $$ are the co-state variables for the dynamics of $$ E_{t}^{P} ,E_{t}^{T} $$ and $$ \tilde{E}_{t} $$ at time *t*, respectively. Using $$ E_{t} = E_{t}^{P} + E_{t}^{T} , $$ the first-order optimality conditions are:

10 $$ \frac{{\partial {\rm H}}}{{\partial a_{t} }} = - c_{t}^{ - IIA} A_{1} e^{{ - r_{A} t}} a_{t}^{{\theta_{a} - 1}} (1 - m_{t} )\gamma_{0} e^{{ - r_{\gamma } t}} - \left[ {\beta_{0} \lambda_{Pt} + (1 - \beta_{0} )\lambda_{Tt} } \right](1 - m_{t} )\gamma_{0} e^{{ - r_{\gamma } t}} Y_{0} e^{gt} = 0, $$

11 $$ \begin{aligned} \frac{{\partial {\rm H}}}{{\partial m_{t} }} & = c_{t}^{ - IIA} \left[ {\left( {G_{0} e^{{ - r_{F} t}} + \frac{1}{{\theta_{a} }}A_{1} e^{{ - r_{A} t}} a_{t}^{{\theta_{a} }} } \right) - m_{t}^{{\theta_{m} - 1}} H_{1} e^{{ - r_{R} t}} - H_{0} } \right]\gamma_{0} e^{{ - r_{\gamma } t}} \\ & \quad - \left[ {\beta_{0} \lambda_{Pt} + (1 - \beta_{0} )\lambda_{Tt} } \right](1 - a_{t} )\gamma_{0} e^{{ - r_{\gamma } t}} Y_{0} e^{gt} = 0, \\ \end{aligned} $$

12 $$ r\lambda_{Pt} - \dot{\lambda }_{Pt} = \frac{{\partial {\rm H}}}{{\partial E_{t}^{P} }} = \tilde{\lambda }_{t} /Tlag, $$

13 $$ r\lambda_{Tt} - \dot{\lambda }_{Tt} = \frac{{\partial {\rm H}}}{{\partial E_{t}^{T} }} = \tilde{\lambda }_{t} /Tlag - \beta_{1} \lambda_{Tt} , $$

14 $$ r\tilde{\lambda }_{t} - \dot{\tilde{\lambda }}_{t} = - dc_{t}^{ - IIA} (Y_{0} e^{gt} )^{ - 1} - \tilde{\lambda }_{t} /Tlag. $$

Upon defining $P_{t} \equiv - c_{t}^{IIA} \left[ {\beta_{0} \lambda_{Pt} + (1 - \beta_{0} )\lambda_{Tt} } \right]Y_{0} e^{gt} ,$ (10) and (11) yield (2) and (3). For purposes of calculating our simple rule for the optimal price of carbon only, we suppose that along a steady-growth path, *c* is approximately constant. In our baseline calibrations presented in Fig. 1 and Table 2, *c* varies by at most 3% over time. We note that for *IIA* = 0, our equilibrium expressions for climate policy are exact. Hence, (14) gives $\tilde{\lambda }_{t} Y_{0} e^{gt} \cong - \frac{1}{r + 1/Tlag}dc^{ - IIA}$ and (12) and (13) give $- \lambda_{Pt} Y_{0} e^{gt} c^{IIA} = \frac{1}{r}\left( {\frac{1}{1 + r \times Tlag}} \right)d$ and $- \lambda_{Tt} Y_{0} e^{gt} c^{IIA} = \frac{1}{{r + \beta_{1} }}\frac{1}{1 + r \times Tlag}d.$ Hence, $\tau = \left( {\frac{{\beta_{0} }}{r} + \frac{{1 - \beta_{0} }}{{r + \beta_{1} }}} \right)\frac{1}{1 + r \times Tlag}d,$ so $P_{t} = \tau Y_{0} e^{gt}$ is given by (7). The transition time condition is that the marginal cost of the last ton of fossil fuel equals the marginal cost of renewables at full decarbonisation, $H_{0} + m_{T}^{{\theta_{m} - 1}} H_{1} e^{{ - r_{R} T}} = G_{0} e^{{ - r_{F} T}} + \frac{1}{{\theta_{a} }}A_{1} e^{{ - r_{A} T}} a_{T}^{{\theta_{a} }} + (1 - a_{T} )P_{T}$ with $m_{T} = 1.$

In case of full sequestration takes place before the energy mix has fully eliminated replaced fossil fuel by renewables, $$ a_{T} = 1 $$ for $$ T' \le t < T, $$ the transition time for full sequestration $$ T' $$ satisfies $$ A_{1} e^{{ - r_{A} T'}} = \tau Y_{0} e^{gT'} $$ and for full decarbonisation from $$ G_{0} e^{{ - r_{F} T}} + \frac{1}{{\theta_{A} }}A_{1} e^{{ - r_{A} T}} a_{T}^{{\theta_{a} - 1}} = H_{0} + H_{1} e^{{ - r_{R} T}} $$ with $$ a_{T} = 1, $$ so $$ T' = \frac{1}{{r_{A} + g}}\ln \left( {\frac{{A_{1} }}{{\tau Y_{0} }}} \right) < T $$ and *T* from $$ G_{0} e^{{ - r_{F} T}} + \frac{1}{{\theta_{a} }}A_{1} e^{{ - r_{A} T}} = H_{0} + H_{1} e^{{ - r_{R} T}} . $$ □

## Appendix 2: Pricing Carbon Under Hyperbolic Discounting

With hyperbolic discounting the Hamiltonian for this problem is

$$ \begin{aligned}& {\rm H}^{Hyperbolic}  \equiv \left( {\frac{1}{1 + \iota t}} \right)^{ - \rho /\iota } \frac{{e^{(1 - IIA)gt} }}{1 - IIA} \\ & \quad \times \left[ {1 - d\tilde{E}_{t} - \left( {G_{0} e^{{ - r_{F} t}} + \frac{1}{{\theta_{a} }}A_{1} e^{{ - r_{A} t}} a_{t}^{{\theta_{a} }} } \right)(1 - m_{t} )\gamma_{0} e^{{ - r_{\gamma } t}} - \left( {H_{0} m_{t} + \frac{1}{{\theta_{m} }}m_{t}^{{\theta_{m} }} H_{1} e^{{ - r_{R} t}} } \right)\gamma_{0} e^{{ - r_{\gamma } t}} } \right]^{1 - IIA} \\ & \quad { + }\,\lambda_{P} \beta_{0} (1 - a_{t} )(1 - m_{t} )\gamma_{0} e^{{ - r_{\gamma } t}} Y_{0} e^{gt} + \lambda_{T} \left\{ {(1 - \beta_{0} )(1 - a_{t} )(1 - m_{t} )\gamma_{0} e^{{ - r_{\gamma } t}} Y_{0} e^{gt} - \beta_{1} E_{t}^{T} } \right\} \\ & \quad + \tilde{\lambda }(E_{t}^{P} + E_{t}^{T} - \tilde{E}_{t} )/Tlag. \\ \end{aligned} $$

where $$ \lambda_{Pt} ,\lambda_{Tt} $$ and $$ \tilde{\lambda }_{t} $$ are the (discounted) co-states for the dynamics of $$ E_{t}^{P} ,E_{t}^{T} $$ and $$ \tilde{E}_{t} $$ at time *t*, respectively. Using $$ E_{t} = E_{t}^{P} + E_{t}^{T} , $$ the first-order optimality conditions are:

15 $$ \frac{{\partial {\rm H}^{Hyperbolic} }}{{\partial a_{t} }} = - c_{t}^{ - IIA} A_{1} e^{{ - r_{A} t}} a_{t}^{{\theta_{a} - 1}} (1 - m_{t} )\gamma_{0} e^{{ - r_{\gamma } t}} - \left[ {\beta_{0} \lambda_{Pt} + (1 - \beta_{0} )\lambda_{Tt} } \right](1 - m_{t} )\gamma_{0} e^{{ - r_{\gamma } t}} Y_{0} e^{gt} = 0, $$

16 $$ \begin{aligned} \frac{{\partial {\rm H}^{Hyperbolic} }}{{\partial m_{t} }} & = c_{t}^{ - IIA} \left[ {\left( {G_{0} e^{{ - r_{F} t}} + \frac{1}{{\theta_{a} }}A_{1} e^{{ - r_{A} t}} a_{t}^{{\theta_{a} }} } \right) - m_{t}^{{\theta_{m} - 1}} H_{1} e^{{ - r_{R} t}} - H_{0} } \right]\gamma_{0} e^{{ - r_{\gamma } t}} \\ & \quad - \left[ {\beta_{0} \lambda_{Pt} + (1 - \beta_{0} )\lambda_{Tt} } \right](1 - a_{t} )\gamma_{0} e^{{ - r_{\gamma } t}} Y_{0} e^{gt} = 0, \\ \end{aligned} $$

17 $$ - \dot{\lambda }_{Pt} = \frac{{\partial {\rm H}^{Hyperbolic} }}{{\partial E_{t}^{P} }} = \tilde{\lambda }_{t} /Tlag, $$

18 $$ - \dot{\lambda }_{Tt} = \frac{{\partial {\rm H}^{Hyperbolic} }}{{\partial E_{t}^{T} }} = \tilde{\lambda }_{t} /Tlag - \beta_{1} \lambda_{Tt} , $$

19 $$ - \dot{\tilde{\lambda }}_{t} = \frac{{\partial {\rm H}^{Hyperbolic} }}{{\partial \tilde{E}_{t} }} = - dc_{t}^{ - IIA} e^{ - g(IIA - 1)t} (1 + \iota t)^{ - \rho /\iota } - \tilde{\lambda }_{t} /Tlag. $$

Again, for purposes of deriving our simple rule for the optimal price of carbon only, we suppose that along a steady growth path, *c* is approximately constant or that *IIA* = 0. Hence, (19) gives $\tilde{\lambda }_{t} = - c^{ - IIA} d\,\iota^{ - 1} (1 + \iota t)^{1 - \rho /\iota } e^{\omega } E_{{^{{\tilde{\rho }/\iota }} }} \left( {\omega_{t} } \right)$ where $\omega_{t} = \left( {Tlag^{ - 1} - (IIA - 1)g} \right)\left( {\iota^{ - 1} + t} \right)$ and $E_{n} (x) = \int_{1}^{\infty } {\frac{{e^{ - xt} }}{{t^{n} }}dt}$ is the generalised exponential integral function. Equations (17) and (18) give $\lambda_{Pt} = - c^{ - IIA} d\left( {\frac{1}{1 + \iota t}} \right)^{\rho /a} e^{{\frac{g(IIA - 1)}{\iota }}} \big( \iota^{ - 1} + t \big)\Big\{ E_{{^{\rho /\iota } }} \big( g(IIA - 1)(\iota^{ - 1} + t) \big) - e^{{l^{ - 1} \big( \iota^{ - 1} + t \big)}} E_{{^{\rho /\iota } }} \big( \big[ g(IIA - 1) + Tlag^{ - 1}  \big](\iota^{ - 1} + t) \big) \Big\}$ and $\lambda_{Tt} = - c^{ - IIA} d\left( {\frac{1}{1 + \iota t}} \right)^{\rho /\iota } e^{{\frac{g(IIA - 1)}{\iota }}} \frac{{\left( {\iota^{ - 1} + t} \right)}}{{1 - Tlag \times \beta_{1} }} \times \left\{ {e^{{\beta_{1} \left( {a^{ - 1} + t} \right)}} E_{{^{\rho /\iota } }} \left( {\left[ {g(IIA - 1) + \beta_{1} } \right](\iota^{ - 1} + t)} \right)} \right.\, - \left. {e^{{l^{ - 1} \left( {\iota^{ - 1} + t} \right)}} E_{{^{\rho /a} }} \left( {\left[ {g(IIA - 1) + Tlag^{ - 1} } \right](\iota^{ - 1} + t)} \right)} \right\}.$

We achieve these results and avoid potential problems of multiplicity by solving the model for a finite horizon *H* and taking the limit *H* → ∞.

Defining $P_{t} \equiv - c_{t}^{IIA} \left[ {\beta_{0} \lambda_{Pt} + (1 - \beta_{0} )\lambda_{Tt} } \right]Y_{0} e^{gt}$ as before, our simple rule for the optimal price of carbon becomes (8). Taking the limit $\iota$  → 0, gives (7).

To obtain the feedback Nash equilibrium without commitment, we have to take into account the resetting of carbon prices in each period. In our simple framework, the only time-varying determinant of the carbon price is exogenously growing GDP. We can, therefore, simply evaluate the carbon price of (8) at time *t* = 0 and substitute $Y(t)$ for $Y_{0}$ to obtain (8′).

In a more general model, it is much more difficult to calculate the feedback or subgame-perfect Nash equilibrium as one would allow for capital stock dynamics and more general equilibrium interactions of the economic and climate system. This is even so for the relatively straight-forward case of the special assumption of logarithmic utility, Cobb-Douglas production function, 100% depreciation of capital each period, exponential damages to TFP and a linear carbon cycle and used by Golosov et al. (2014) and quasi-hyperbolic discounting in discrete time which has exponential discounting for all future periods and an additional parameter to bias up the welfare weight of the present by more than all future periods (Gerlagh and Liski 2018). The analysis is more complicated with more general functional forms and generalised hyperbolic discounting (Iverson and Karp 2018).

## Appendix 3: Further Details on the Benchmark Calibration

The economic drivers of climate policy consist of initial fossil fuel and renewable energy costs, *G*_0_, *H*_0_, and *H*_1_, which are calibrated to give current energy cost shares of 7% of GDP and the additional cost of 5.6% of GDP for full decarbonisation following DICE. The rate of directed technical change of rate of 1.3% per year is chosen to match the cost of 1.6% of GDP for full decarbonisation in 100 years, again based on DICE. The cost of carbon-based energy increases by 0.1% per year to capture resource scarcity and match baseline emissions scenarios of the EMF-22 (Nordhaus 2015). The cost of full sequestration is calibrated to initial 20% of GDP, falls at the rate of non-carbon technologies, and decreases to 5.7% of GDP in 100 years. Given that there is large uncertainty around the technological prospects of CCS and other abatement technologies, we conduct sensitivity analyses around them in Sect. 7. Initial GDP is 73 T$ and energy use per unit of GDP of 0.14 GtC/T$ is calibrated to match current yearly emissions of 10 GtC. We keep emissions intensity itself constant as we capture the adoptions of carbon-free technologies endogenously in *a*_*t*_ and *m*_*t*_. The flow damage of the stock of carbon in the atmosphere of 0.019 $/tC which equals 0.5% of GDP at current levels of atmospheric carbon.

The calibration of the climate module to the model of Joos et al. (2013) follows except the calibration of the lag structure which deviates from the original model. Here we use the finding of Caldeira and Ricke (2014) who find that maximum peak warming occurs in the median after 10 years in the model of Joos et al. (2013). Together with the fact current and committed warming currently are 1 °C and 1.3 °C, respectively, we have $Tlag = - 10/\ln \left[ {4.33(1 - x)} \right],$ with *x* the percentage of peak warming occurring after 10 years. We set *x* = 99% and *Tlag* = 3.2 years.

## Appendix 4: Pricing carbon under declining absorption rates

With a time-dependent absorption rate the Hamiltonian with climate feedback for this problem is

$$ \begin{aligned} {\rm H}^{c.f.} & \equiv \frac{1}{1 - IIA}\left[ 1 - d\tilde{E}_{t} - \left( {H_{0} m_{t} + \frac{1}{{\theta_{m} }}m_{t}^{{\theta_{m} }} H_{1} e^{{ - r_{R} t}} } \right)\gamma_{0} e^{{ - r_{\gamma } t}}  \right.\\ &\quad \left.  - \left( {G_{0} e^{{ - r_{F} t}} + \frac{1}{{\theta_{a} }}A_{1} e^{{ - r_{A} t}} a_{t}^{{\theta_{a} }} } \right)(1 - m_{t} )\gamma_{0} e^{{ - r_{\gamma } t}}  \right]^{1 - IIA} \\ & \quad { + }\,\tilde{\lambda }(E_{t}^{P} + E_{t}^{T} - \tilde{E}_{t} )/Tlag\;{ + }\;\lambda_{P} \beta_{0} (1 - a_{t} )(1 - m_{t} )\gamma_{0} e^{{ - r_{\gamma } t}} Y_{0} e^{gt} \\ & \quad +\,\lambda_{T} \left( {(1 - \beta_{0} )(1 - a_{t} )(1 - m_{t} )\gamma_{0} e^{{ - r_{\gamma } t}} Y_{0} e^{gt} - \beta_{1} (1 - \alpha \;t)E_{t}^{T} } \right). \\ \end{aligned} $$

where $$ \lambda_{Pt} ,\lambda_{Tt} $$ and $$ \tilde{\lambda }_{t} $$ are the (discounted) co-states for the dynamics of $$ E_{t}^{P} ,E_{t}^{T} $$ and $$ \tilde{E}_{t} $$ at time *t*, respectively. Using $$ E_{t} = E_{t}^{P} + E_{t}^{T} , $$ the first-order optimality conditions are:

20 $$ \frac{{\partial {\rm H}^{c.f.} }}{{\partial a_{t} }} = - c_{t}^{ - IIA} A_{1} e^{{ - r_{A} t}} a_{t}^{{\theta_{a} - 1}} (1 - m_{t} )\gamma_{0} e^{{ - r_{\gamma } t}} - \left[ {\beta_{0} \lambda_{Pt} + (1 - \beta_{0} )\lambda_{Tt} } \right](1 - m_{t} )\gamma_{0} e^{{ - r_{\gamma } t}} Y_{0} e^{gt} = 0, $$

21 $$ \begin{aligned} \frac{{\partial {\rm H}^{c.f.} }}{{\partial m_{t} }} & = c_{t}^{ - IIA} \left[ {\left( {G_{0} e^{{ - r_{F} t}} + \frac{1}{{\theta_{a} }}A_{1} e^{{ - r_{A} t}} a_{t}^{{\theta_{a} }} } \right) - m_{t}^{{\theta_{m} - 1}} H_{1} e^{{ - r_{R} t}} - H_{0} } \right]\gamma_{0} e^{{ - r_{\gamma } t}} \\ & \quad - \left[ {\beta_{0} \lambda_{Pt} + (1 - \beta_{0} )\lambda_{Tt} } \right](1 - a_{t} )\gamma_{0} e^{{ - r_{\gamma } t}} Y_{0} e^{gt} = 0, \\ \end{aligned} $$

22 $$ - \dot{\lambda }_{Pt} = \frac{{\partial {\rm H}^{c.f.} }}{{\partial E_{t}^{P} }} = \tilde{\lambda }_{t} /Tlag, $$

23 $$ - \dot{\lambda }_{Tt} = \frac{{\partial {\rm H}^{c.f.} }}{{\partial E_{t}^{T} }} = \tilde{\lambda }_{t} /Tlag - \beta_{1} (1 - \alpha \,t)\lambda_{Tt} , $$

24 $$ - \dot{\tilde{\lambda }}_{t} = \frac{{\partial {\rm H}^{c.f.} }}{{\partial \tilde{E}_{t} }} = - dc_{t}^{ - IIA} e^{ - g(IIA - 1)t} - \tilde{\lambda }_{t} /Tlag. $$

Again, for purposes of deriving our simple rule for the optimal price of carbon only, we suppose that along a steady growth path, *c* is approximately constant or that *IIA* = 0. Hence, (24) gives $$ \tilde{\lambda }_{t} Y_{0} e^{gt} \cong - \frac{1}{r + 1/Tlag}dc^{ - IIA} . $$ Equations (17) and (18) give $$ \lambda_{Pt} Y_{0} e^{gt} c^{IIA} = - \frac{1}{r}\left( {\frac{1}{1 + r \times Tlag}} \right)d $$$$ {\text{and}}\;\;\lambda_{Tt} Y_{0} e^{gt} c^{IIA} = \frac{d}{1 + r \times Tlag}\frac{2}{{\sqrt {2\alpha \beta_{1} } }}\varPhi \left[ { - \frac{{r + \beta_{1} (1 - \alpha t)}}{{\sqrt {2\alpha \beta_{1} } }}} \right] $$ with $$ \varPhi \left[ x \right] = e^{{ - x^{2} }} \int_{0}^{x} {e^{{y^{2} }} dy} $$ the Dawson integral of *x*. Defining $$ P_{t} \equiv - c_{t}^{IIA} \left[ {\beta_{0} \lambda_{Pt} + (1 - \beta_{0} )\lambda_{Tt} } \right]Y_{0} e^{gt} , $$ the simple rule for the optimal price of carbon with declining absorption can be evaluated parametrically. *a*_t_ and *m*_t_ follow from (20) and (21) and are identical to the expressions given in (1) and (2).

## Appendix 5: Fossil Fuel Extraction Costs That Rise as Reserves Fall, Sequestration Costs That Rise and Renewable Costs That Fall as Cumulative Use Rises

Here we consider the case when fossil fuel extraction get more costly if reserves fall, so costs increase with cumulative fossil fuel use: $G(S_{t} ) = G_{0} e^{{ - r_{F} t}} \left[ {1 + e\frac{{S_{0} - S_{t} }}{{S_{0} }}} \right],$ with *e* > 0 and *S*_0_ and *S*_*t*_ denoting initial fossil fuel reserves at time zero and *t*, respectively. Fossil fuel reserves decline according to the depletion equation $\dot{S} = - (1 - m)\gamma Y$ with *S*_0_ given. Cumulative fossil fuel use is *S*_0_ – *S*_*T*_. We also let costs for sequestration rise as more carbon is sequestrated and reservoir capacity has shrunk for geological or NIMBY reasons, so cost of sequestration can be specified as $\frac{1}{{\theta_{a} }}A_{1} e^{{ - r_{A} t}} a_{t}^{{\theta_{a} }} V_{t}^{{\beta_{2} }} ,$ with *β*_2_ > 0, where the stock of sequestrated carbon evolves according to $\dot{V} = aF = a(1 - m)\gamma Y.$ We let renewable energy production gets cheaper with learning by doing, one can specify the cost of mitigation as $\frac{1}{{\theta_{m} }}m_{t}^{{\theta_{m} }} H_{1} e^{{ - r_{R} t}} B_{t}^{{ - \beta_{3} }}$ with *β*_3_ > 0, where the stock of accumulated knowledge about renewable energy production evolves according to $\dot{B} = R = m\gamma Y.$ We then get the following generalised rules for climate policy.

**Result A1:** Our simple rule for the optimal carbon price is given by (7) in Result 1. The time paths for the optimal sequestration and share of renewables in the energy mix are

25 $$ a_{t} = \left( {\frac{{\tau_{t} - \theta_{Vt} }}{{A_{1} V_{t}^{{\beta_{2} }} }}} \right)^{{\varepsilon_{a} }} e^{{r_{A} \varepsilon_{a} t}} ,\quad 0 \le t < \hbox{min} [T,T'], $$

26 $$ \begin{aligned} m_{t} = \left( {\frac{{G_{0} e^{{ - r_{F} t}} \left[ {1 + e\left( {1 - S_{t} /S_{0} } \right)} \right] + \frac{1}{{\theta_{a} }}A_{0} e^{{ - r_{A} t}} a_{t}^{{\theta_{a} }} V_{t}^{{\beta_{2} }} - H_{0} + X_{t} }}{{H_{1} e^{{ - r_{R} t}} B_{t}^{{ - \beta_{3} }} }}} \right)^{{\varepsilon_{m} }} , \hfill \\ \quad \quad 0 \le t < T{\text{ and }}m(t) = 1,\;t \ge T, \hfill \\ \end{aligned} $$

where $$ X_{t} \equiv \tau_{t} + \theta_{St} + \theta_{Bt} - (\tau_{t} - \theta_{Vt} )a_{t} $$ and $$ \theta_{S} (t), $$$$ \theta_{V} (t) $$ and $$ \theta_{B} (t) $$ are the scarcity value of in situ fossil fuel, the social cost of sequestrating an additional unit of carbon, and the social benefit of learning by doing in renewable energy production at time *t*, respectively.

### *Proof*

The Hamiltonian function is

$$ \begin{aligned} {\rm H}^{extended} & \equiv \frac{1}{1 - IIA}c_{t}^{1 - IIA} { + }\lambda_{Pt} \beta_{0} (1 - a_{t} )(1 - m_{t} )\gamma_{0} e^{{ - r_{\gamma } t}} + \lambda_{Tt} \left( {(1 - \beta_{0} )(1 - a_{t} )(1 - m_{t} )\gamma_{0} e^{{ - r_{\gamma } t}} - \beta_{1} E_{t}^{T} } \right) \\ & \quad + \tilde{\lambda }_{t} (E_{t} - \tilde{E}_{t} )/Tlag + \left[ { - \lambda_{St} (1 - m_{t} ) + \lambda_{Vt} a_{t} (1 - m_{t} ) + \lambda_{Bt} m_{t} } \right]\gamma_{0} e^{{ - r_{\gamma } t}} Y_{0} e^{gt} , \\ \end{aligned} $$

where $\tilde{\lambda },\lambda_{S} ,\lambda_{V}$ and $\lambda_{B}$ are the co-states for $\tilde{E},$*S*, *V* and *B* respectively, and

$$ c_{t} = 1 - d\tilde{E}_{t} - \left( {G_{0} e^{{ - r_{F} t}} \left[ {1 + e\frac{{S_{0} - S_{t} }}{{S_{0} }}} \right] + \frac{1}{{\theta_{a} }}A_{0} e^{{ - r_{A} t}} a_{t}^{{\theta_{a} }} V_{t}^{{\beta_{2} }} } \right)(1 - m_{t} )\gamma_{0} e^{{ - r_{\gamma } t}} - H_{0} m_{t} \gamma_{0} e^{{ - r_{\gamma } t}} $$

$- \frac{1}{{\theta_{m} }}m_{t}^{{\theta_{m} }} H_{1} e^{{ - r_{R} t}} B_{t}^{{ - \beta_{3} }} \gamma_{0} e^{{ - r_{\gamma } t}} .$ The first-order optimality conditions are (12), (13), (14),

27 $$ \begin{aligned} \frac{{\partial {\rm H}^{extended} }}{{\partial a_{t} }} & = - c_{t}^{ - IIA} A_{0} e^{{ - r_{A} t}} a_{t}^{{\theta_{a} - 1}} V_{t}^{{\beta_{2} }} (1 - m_{t} )\gamma_{0} e^{{ - r_{\gamma } t}} \\ & \quad - \left[ {\beta_{0} \lambda_{Pt} + (1 - \beta_{0} )\lambda_{Tt} } \right](1 - m_{t} )\gamma_{0} e^{{ - r_{\gamma } t}} + \lambda_{Vt} (1 - m_{t} )\gamma_{0} e^{{ - r_{\gamma } t}} Y_{0} e^{gt} = 0, \\ \end{aligned} $$

28 $$ \begin{aligned} \frac{{\partial {\rm H}^{extended} }}{{\partial m_{t} }} & = c_{t}^{ - IIA} \gamma_{0} e^{{ - r_{\gamma } t}} \left( G_{0} e^{{ - r_{F} t}} \left[ {1 + e\frac{{S_{0} - S_{t} }}{{S_{0} }}} \right] + \frac{1}{{\theta_{a} }}A_{0} e^{{ - r_{A} t}} a_{t}^{{\theta_{a} }} V_{t}^{{\beta_{2} }} - m_{t}^{{\theta_{m} - 1}}\right. \\ & \quad  H_{1} e^{{ - r_{R} t}} B_{t}^{{ - \beta_{3} }}  - H_{0} \left. { + P_{t} (1 - a_{t} )} \right) - (\lambda_{V} a - \lambda_{S} - \lambda_{B} )\gamma_{0} e^{{ - r_{\gamma } t}} Y_{0} e^{gt} = 0, \\ \end{aligned} $$

29 $$ r\lambda_{St} - \dot{\lambda }_{St} = \frac{{\partial {\rm H}^{extended} }}{{\partial S_{t} }} = c_{t}^{ - IIA} G_{0} e^{{ - r_{F} t}} \frac{e}{{S_{0} }}(1 - m_{t} )\gamma_{0} e^{{ - r_{\gamma } t}} , $$

30 $$ r\lambda_{Vt} - \dot{\lambda }_{Vt} = \frac{{\partial {\rm H}^{extended} }}{{\partial V_{t} }} = - \frac{{\beta_{2} }}{{\theta_{a} }}A_{0} e^{{ - r_{A} t}} a_{t}^{{\theta_{a} }} V_{t}^{{\beta_{2} - 1}} (1 - m_{t} )\gamma_{0} e^{{ - r_{\gamma } t}} c_{t}^{IIA} , $$

31 $$ r\lambda_{Bt} - \dot{\lambda }_{Bt} = \frac{{\partial {\rm H}^{extended} }}{{\partial B_{t} }} = \frac{{\beta_{3} }}{{\theta_{m} }}m_{t}^{{\theta_{m} }} H_{1} e^{{ - r_{R} t}} B_{t}^{{ - \beta_{3} - 1}} \gamma_{0} e^{{ - r_{\gamma } t}} c_{t}^{IIA} . $$

Defining $$ P_{t} = \tau Y_{0} e^{gt} $$ and $$ \tau_{t} \equiv - c_{t}^{IIA} \left[ {\beta_{0} \lambda_{Pt} + (1 - \beta_{0} )\lambda_{Tt} } \right]Y_{0} e^{gt} $$ as in proof of Result 1, we get (7) from (12), (13) and (14). With $$ \theta_{St} \equiv c_{t}^{IIA} \lambda_{St} Y_{0} e^{gt} , $$$$ \theta_{Vt} \equiv - c_{t}^{IIA} \lambda_{Vt} Y_{0} e^{gt} $$ and $$ \theta_{Bt} \equiv c_{t}^{IIA} \lambda_{Bt} Y_{0} e^{gt} , $$ we see that Eq. (23) yields

32 $$ G_{0} e^{{ - r_{F} t}} \left[ {1 + e\frac{{S_{0} - S_{t} }}{{S_{0} }}} \right] + \frac{1}{{\theta_{a} }}A_{0} e^{{ - r_{A} t}} a_{t}^{{\theta_{a} }} V(t)^{{\beta_{2} }} - m_{t}^{{\theta_{m} - 1}} H_{1} e^{{ - r_{R} t}} B_{t}^{{ - \beta_{3} }} - H_{0} { = } - \tau (1 - a_{t} ) - (\theta_{V} a + \theta_{S} + \theta_{B} ). $$

Equation (32) can be rewritten as (26). Similarly, Eq. (15) gives

33 $$ A_{0} e^{{ - r_{A} t}} a_{t}^{{\theta_{a} - 1}} V_{t}^{{\beta_{2} }} { = }\tau - \theta_{V} $$

which gives (25). Equations (29)–(31) give the dynamics of the scarcity rent on fossil fuel (*RFF*), the social cost of sequestration (*SCS*) and the social benefit of learning by doing (*SBL*):

34 $$ \dot{\theta }_{St} = (r - g)\theta_{St} - G_{0} e^{{ - r_{F} t}} \frac{e}{{S_{0} }}(1 - m_{t} )\gamma_{0} e^{{ - r_{\gamma } t}} Y_{0} e^{gt} , $$

35 $$ \dot{\theta }_{Vt} = r\theta_{Vt} - \frac{{\beta_{2} }}{{\theta_{a} }}A_{0} e^{{ - r_{A} t}} a_{t}^{{\theta_{a} }} V_{t}^{{\beta_{2} - 1}} (1 - m_{t} )\gamma_{0} e^{{ - r_{\gamma } t}} Y_{0} e^{gt} , $$

36 $$ \dot{\theta }_{Bt} = (r - g)\theta_{Bt} - \frac{{\beta_{3} }}{{\theta_{m} }}m_{t}^{{\theta_{m} }} H_{0} e^{{ - r_{R} t}} B_{t}^{{ - \beta_{3} - 1}} \gamma_{0} e^{{ - r_{\gamma } t}} Y_{0} e^{gt} . $$

□

This extended back-of-the-envelope IAM needs to solve (34)–(36) together (25)–(26), the depletion equation $\dot{S} = - (1 - m)\gamma_{0} e^{{ - r_{\gamma } t}} Y_{0} e^{gt} ,$ the sequestration sink cost equation $\dot{V} = a(1 - m)\gamma_{0} e^{{ - r_{\gamma } t}} Y_{0} e^{gt} ,$ and the learning-by-doing equation $\dot{B} = m\gamma_{0} e^{{ - r_{\gamma } t}} Y_{0} e^{gt}$ as a two-point-boundary value problem with predetermined values for *S*(0), *V*(0) and *B*(0).

Without the ability to capture the effects of scarcity to deposit sequestered carbon in and the benefits of renewable innovation, there are three market failures now, which need three corrections. The first one is to price carbon at the social cost of carbon (*SCC*), which is the present discounted value of all future marginal damages from burning one unit of fossil fuel today. The second one is to subsidise renewable energy use at the social benefit of learning by doing in renewable energy production (*SBL*), which from (36) amounts to the present discounted value of all future reductions in the cost of renewable energy production from producing one unit of renewable energy today. The third one is a tax on *CCS* to allow for the increasing costs of sequestration as more reservoirs are used up. This tax is set to the social cost of sequestration (*SCS*), which from (35) equals the present value of all future increases in sequestration costs resulting from sequestrating one unit of carbon today. In contrast, the scarcity rent of fossil fuel (*RFF*) is from (34) the present discounted value of all future increases in extraction costs resulting from depleting one unit of fossil fuel today. The *RFF* is internalised by fossil producers and requires no government action.

It follows from Eq. (26) that a bigger share of renewable energy is used if the scarcity rent on fossil fuel, the social benefit of learning in renewable energy production, and, to the extent that it is used, the social cost of sequestration is high. In other words, the share of renewables in the energy mix increases in *RFF*+*SCC*+*SBL* and, to the extent that sequestration takes place, decreases in *SCC*-*SCS*. As in Eq. (1) of Result 1, it also increases in the costs of extracting fossil fuel and sequestrating carbon emissions. Equation (25) extends Eq. (2) and shows that the fraction of carbon that is sequestrated increases in the difference between the price of carbon and the social cost of sequestration, i.e., it increases in *SCC*–*SCS*.

## Footnotes

1 We refer to the papers of Gerlagh and Liski (2018) and Iverson and Karp (2018) for time-consistent (technically subgame-perfect Markov equilibrium) solutions to the difficult problem of deriving welfare-maximising climate policies under quasi-hyperbolic discounting in general equilibrium models with capital formation. See also Fujii and Karp (2008).

2 An Excel sheet is available for those who wish to examine the effect of varying assumptions and parameters values on optimal climate policies.

3 In contrast, one can examine breakthrough renewable energy which comes in as a perfect substitute for fossil fuel only once it has gained a cost advantage and signals the abrupt end of the carbon era (e.g., Rezai and van der Ploeg 2016). Somewhat unrealistically, the only lever of climate policy was thus the end of the carbon era (not the emission ratio) and thus cumulative emissions and peak warming. However, partial carbon reduction where mitigation and abatement are used alongside each other is clearly more realistic, so this together with the optimal transition times for the various energy phases is what we will introduce in an easy-to-understand, back-of-the-envelope IAM.

4 While early contributions focused the effect of climate policy on stranded natural assets, i.e., the amount of fossil fuel to be abandoned in situ (McGlade and Ekins 2015; van der Ploeg and Rezai 2017), recent studies include effects of policy on stranded physical and financial assets and nation states (Manley et al. 2017; Baldwin et al. 2018; van der Ploeg and Rezai 2018). See also Karp and Rezai (2018) effects on asset prices.

5 Energy modelling is more reduced in DICE. With *H*_0_ = *G*_0_ = 0 our energy sector is equivalent to that of DICE. By including the unit cost component *H*_0_ and *G*_0_ we are able to capture cost innovations such as the shale gas revolution which alter the energy mix and shift climate policy.

6 Nordhaus (2013) sets *θ*_*m*_ = 2.8 in which case the carbon price elasticity of mitigation is *ε*_*m*_ = 0.55. DICE models the cost of renewable energy in excess of fossil energy, assuming implicitly a fixed baseline (i.e. fossil) energy share of GDP. We account for the cost of energy generation explicitly.

7 For the iso-elastic utility function, it equals both the coefficient of relative risk aversion and the inverse of the elasticity of intertemporal substitution.

8 The carbon price in Golosov et al. (2014) is the exact social cost of carbon in a model with endogenous manmade capital under the restrictions of logarithmic utility, full depreciation of manmade capital, exponential climate damage, and zero fossil fuel extraction costs.

9 Our IAM with partial sequestration is solved by running Eqs. (1)–(2) with (7) forward in time until there is a time *t* = *T* at which *m*_*t*_ hits 1 from below and *a*_*T*_ < 1. The optimal cumulative carbon emissions (the carbon budget) then simply follow from cumulative use.

10 Our IAM with full sequestration is solved by running Eqs. (1) and (2) using (7) for the optimal carbon price forwards in time until there is a time *t* = *T′* at which *a*_*t*_ hits 1 from below and *m*_*T′*_ < 1. There follows an intermediate phase with fossil fuel with full abatement and renewable energy used together from *T′* to *T* until *m*_*T*_ hits 1 from below. The optimal carbon budget corresponds to cumulative carbon emissions from time 0 to *T′*.

11 Golosov et al. (2014) use a higher figure of 2.379% to allow for a small risk (6.8%) of a 30% catastrophic drop in world GDP at 6 °C. If we used this higher figure, all carbon prices would be 25% higher.

12 In a sample 74% of respondents choose fruit and 26% chocolate when they can have it next week, but people choose 30% fruit and 70% chocolate when they get it today (Read and van Leeuwen 1998); in a different example, 66% choose a low-brow and 34% high-brow video today but next week 37% a low-brow and 63% high-brow video (Read et al.1999). So the self wants to be patient and delay gratification, but actions indicate instant gratification. This is why plans to quit smoking, exercise or lose weight are not followed through (Gruber and Koszegi 2003). People join gym for $75/month but only visit on average 4 times a month, so average cost per visit is $19 instead of $10 on a PAYG basis which seems irrational (Della Vigna and Malmendier, 2004). Similarly, people save less than their target saving (Bernheim, 1992; Choi et al., 2003; Public Agenda, 1997; Angeletos et al., 2001).

13 Seminal applications of declining discount rates to climate change are Karp (2005), Fujii and Karp (2008), Karp and Tsur (2011), Gollier (2012), Gerlagh and Liski (2018), and Karp and Iverson (2018).

14 Our results for exponential discounting are not that different from using DICE-2013R: a RTI of 0.1% gives an initial carbon price of $146/tC compared to our $108/tC. Also, raising the IIA to 2 gives a carbon price of $28/tC just like our back-on-the-envelope IAM.

15 We keep our 1-box model of the temperature dynamics. The calibrated coefficients for (3′) with *K* = 4 are *β*_0_ = 0.217, *β*_11_ = 0.0025, *β*_12_ = 0.0274, *β*_13_ = 0.2323, *β*_21_ = 0.286, *β*_22_ = 0.360, *β*_23_ = 0.352, and *l* = 3.2. See also “Appendix 3”.

16 In particular, we model this by assuming that the dissipation rate decreases linearly in cumulative emissions which themselves can be approximated linearly by time. Our short-cut captures the essence of positive feedback mechanism of Millar et al. (2017) while still allowing for an analytical solution presented in “Appendix 4”.

17 Our framework limits international movements to energy, whilst there is no further international trade in goods and services and no international movement of factors of production. This rather radical form of market incompleteness is not very realistic, but it gives tractable results and has been used before in international climate economics (Nordhaus, 2010; Hassler and Krusell, 2012).

18 In assuming a balanced growth path in the calculation of the optimal price of carbon, we ignore the dynamics of capital accumulation as these converge much faster than the carbon cycle. If one were to allow for these dynamics, the subgame-perfect (or feedback) and open-loop Nash equilibrium outcomes would generally differ.

19 Note that country-specific *T*_i_ results from $G_{0} e^{{ - r_{F} T_{i} }} + \theta_{a}^{ - 1} A_{1} e^{{ - r_{A} T_{i} }} a_{{iT_{i} }}^{{\theta_{a} }} + (1 - a_{{iT_{i} }} )P_{{iT_{i} }} = H_{0} + H_{1} e^{{ - r_{R} T_{i} }}$ and the corresponding carbon budget *B*_*i*_ from cumulative use during time 0 and *T*_*i*_. Global transition time is the maximum *T*_i_ and the global carbon budget $\sum\nolimits_{i} {B_{i} } .$

20 We only disaggregate damages and output and keep technological coefficients uniform across regions. A more detailed analysis would have to account for differentials in growth rates and technological progress across regions.

21 If there is inertia between changes in global mean temperature and the stock of atmospheric carbon, the cost-minimising carbon price follows an inverse U-shaped path and grows more slowly than the Hotelling path (Lemoine and Rudik 2017). However, recent insights from atmospheric science indicates that this inertia is very small and this implies that the cost-minimising carbon price follows a Hotelling path. This means that carbon price is much more quickly and more vigorously. .
